# MAJOR TRANSPORT MECHANISMS OF PYRETHROIDS IN RESIDENTIAL SETTINGS AND EFFECTS OF MITIGATION MEASURES

**DOI:** 10.1002/etc.2411

**Published:** 2013-10-04

**Authors:** Paul C Davidson, Russell L Jones, Christopher M Harbourt, Paul Hendley, Gregory E Goodwin, Bradley A Sliz

**Affiliations:** † Waterborne EnvironmentalChampaign, Illinois, USA; ‡ Bayer CropScienceResearch Triangle Park, North Carolina, USA; § PhaseraBracknell, Berkshire, United Kingdom

**Keywords:** Pyrethroids, Urban runoff, Field studies

## Abstract

The major pathways for transport of pyrethroids were determined in runoff studies conducted at a full-scale test facility in central California, USA. The 6 replicate house lots were typical of front lawns and house fronts of California residential developments and consisted of stucco walls, garage doors, driveways, and residential lawn irrigation sprinkler systems. Each of the 6 lots also included a rainfall simulator to generate artificial rainfall events. Different pyrethroids were applied to 5 surfaces—driveway, garage door and adjacent walls, lawn, lawn perimeter (grass near the house walls), and house walls above grass. The volume of runoff water from each house lot was measured, sampled, and analyzed to determine the amount of pyrethroid mass lost from each surface. Applications to 3 of the house lots were made using the application practices typically used prior to recent label changes, and applications were made to the other 3 house lots according to the revised application procedures. Results from the house lots using the historic application procedures showed that losses of the compounds applied to the driveway and garage door (including the adjacent walls) were 99.75% of total measured runoff losses. The greatest losses were associated with significant rainfall events rather than lawn irrigation events. However, runoff losses were 40 times less using the revised application procedures recently specified on pyrethroid labels. *Environ Toxicol Chem* 2014;33:52–60. © 2013 The Authors. *Environmental Toxicology and Chemistry* published by Wiley Periodicals, Inc. on behalf of SETAC. This is an open access article under the terms of the Creative Commons Attribution License, which permits use, distribution, and reproduction in any medium, provided the original work is properly cited.

## Introduction

Pyrethroids are a chemical class of insecticides used to control a wide range of pests in urban settings. Compounds applied in residential settings, including pyrethroids, have the potential to be transported in runoff water to street and then to street drains, which empty into urban streams. When present in urban streams, pyrethroids have the potential to affect aquatic invertebrates in water and sediments. Pyrethroid use has especially increased in the early 2000s as a result of the elimination of organophosphate insecticides for urban use. California, USA, is an area where pyrethroids are used extensively and have been detected in urban creek sediments [1–3]. In an urban setting, pyrethroids can be applied to grass, driveways, vertical walls, and garage doors using a range of different formulation types. The Pyrethroid Working Group (a task force of pyrethroid registrants) has sponsored studies on runoff from turf [4], wash-off from building surfaces [5], and wash-off from concrete for different product formulations (C.M. Harbourt, unpublished manuscript). In addition, work performed by others [6–9] has shown the effect of formulation and drying time on wash-off from concrete. There was no overall study, however, to determine which application surfaces are contributing the most to overall transport of pyrethroids from the house lot to street drains.

Mitigation measures to reduce off-site movement have been added by the US Environmental Protection Agency (USEPA) to the federal labels of pyrethroids over the past 5 yr. These measures consist of a number of recommendations, but perhaps the most important is the following: “Other than applications to building foundations, all outdoor applications to impervious surfaces such as sidewalks, driveways, patios, porches and structural surfaces (such as windows, doors and eaves) are limited to spot and crack-and-crevice applications.…” [10]. When these measures were adopted, there was no information of how effective these measures would be in reducing off-site movement.

Therefore, the Pyrethroid Working Group decided to conduct a study to determine important transport pathways and the effectiveness of the mitigation measures. The objectives of the present study were 1) to assess the relative contribution to off-site movement of pyrethroids from a range of possible outdoor residential applications (to pervious surfaces such as turf or shrubs and the soil under them, horizontal impervious surfaces, or vertical building surfaces) and 2) to analyze the effect of the mitigation measures recently adopted by USEPA for reducing off-site transport, in particular the requirement for spot applications on impervious surfaces.

## Materials and Methods

### Basic study design

The study was conducted at a full-scale test facility in central California, USA. Five replicate house lots were typical of front lawns and house fronts of California residential developments and consisted of stucco walls, garage doors, driveways, and residential lawn irrigation sprinkler systems. Each of the 6 lots also included a rainfall simulator to generate artificial rainfall events. Different pyrethroids were applied to 5 surfaces—driveway, garage door and adjacent walls, lawn, lawn perimeter (grass near the house walls), and house walls not adjacent to concrete. The volume of runoff water from each house lot was measured, sampled, and analyzed to determine the mass of pyrethroid lost from each surface. Broadcast applications were made to the lawns once, and perimeter applications were made every 2 mo. Applications to 3 of the house lots were made using the application practices typically used prior to recent label changes, and applications were made to the other 3 house lots according to the revised application procedures (see *Product application procedures*). On the 3 surfaces not affected by the change in application procedures (lawn, lawn perimeter, and house wall not adjacent to concrete) products with contrasting wash-off behavior were used.

### Experimental site

**Figure 1:**
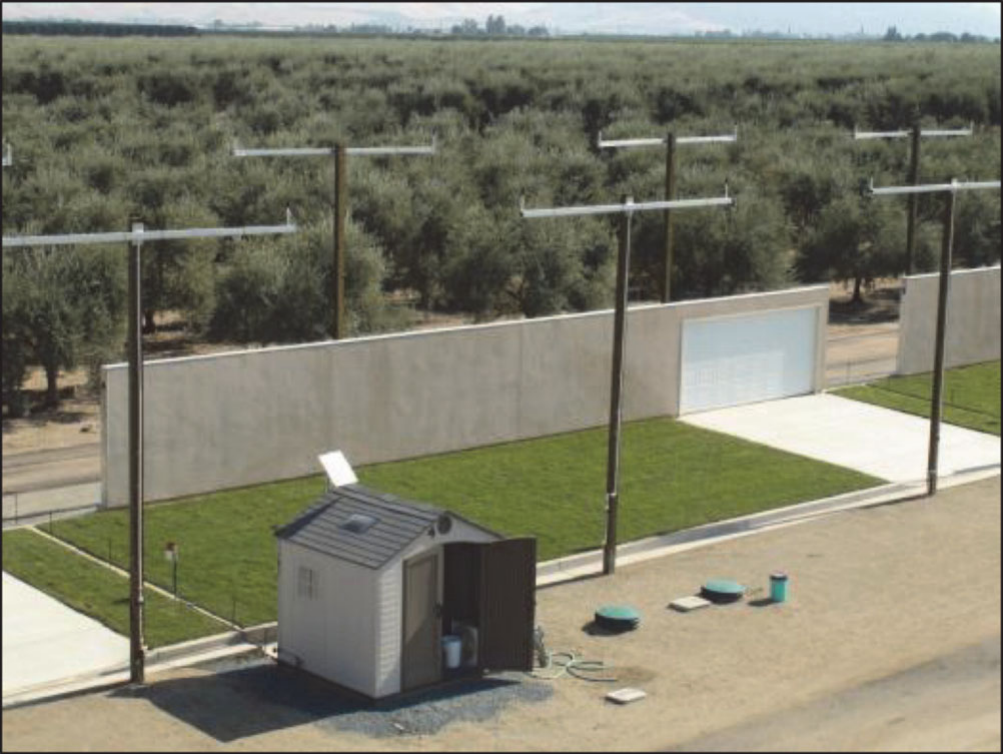
Picture of an individual house lot.

The experimental site was located on an experimental farm in central California near Porterville. This site contained a Tulunga loamy sand soil, which is a preferred soil for building homes in the area. To increase runoff, a clay loam soil with a higher clay content was brought in and applied as a base layer (10–15 cm) on top of the existing soil. The site consisted of 6 identically assembled simulated house lots (a picture of an individual lot is shown in Figure 1). Each house lot was 22.9 m in width, with the length of the house and 2-car garage facade wall measuring 19.8 m. The width of the concrete driveway was 5.5 m, and the distance between the concrete curb and house wall was 6.1 m. The placement of the driveway provided 2 separate sections of grass lawn. The section on the west side of the driveway was approximately 15.8 m wide, and the section on the east side was approximately 1.5 m wide. Both sections of the lawn were treated the same throughout the study. During construction, the lots were graded with driveways sloping (∼6%) from the house to the street, according to normal practice in many suburban areas of California. The garage doors were made of real but fixed and nonoperational painted aluminum garage door panels. House exterior walls were approximately 2.4 m tall, constructed of a sturdy substrate, and covered in typical California polymer-modified stucco using local California contractors following techniques and material arrangements typical of California construction. Particular attention was focused on accurately reproducing the transition area between the stucco wall and slab foundation. There were no overhanging eaves above the walls, and the roof area and back yards were not included in the test site. The 6 lots were side by side in an east–west orientation with the facade walls facing south. In central California, the southwest sides of buildings typically receive more intense direct rain (F. Spurlock, California Department of Pesticide Regulation, Sacramento, California, USA, personal communication).

Professional landscape contractors installed the residential irrigation systems using typical components. The sprinkler heads were arranged in the corners of the larger lawn section (west side of each driveway) and then every 4.0 m along the perimeter of the larger lawn section, giving 8 sprinkler heads. On the small lawn section (east side of each driveway), the sprinkler heads were positioned at the midpoint of each side, giving 4 sprinkler heads.

The rainfall simulator consisted of nozzles on cross-beams mounted on risers located approximately 6.1 m above the ground (so that the drops reach terminal velocity) spaced at 6.1 m intervals just outside the curb and wall. Each nozzle covered a radius of approximately 6.1 m, the same as the depth of the house lots, and produced a random distribution of rain droplets, closely mimicking natural rainfall patterns.

Runoff water from each site flowed down the curb and at the west end of the plot made a 90° turn into a 37.9-L stainless-steel collection basin located below a sampling shed. The sheds were approximately 2.4 m × 2.4 m and housed the runoff collection basin, refrigerator, an Isco 6712 autosampler (placed inside a refrigerator with its temperature monitored by a Campbell Scientific Incorporated [CSI] CS107 temperature probe), CSI CR1000 data-logging system, and various other electronic components. Water from the collection basin was pumped to a 5680-L concrete tank, where it was collected and stored until it could be transported off-site. Each house lot also had a rain gauge and soil moisture and soil temperature probe attached to the CR1000 in the shed. Over the duration of the study, meteorological data were collected for the sampling site by a weather station.

### Product selection

Eight products were selected for use in the study: 2 products with contrasting wash-off behavior for 3 surfaces (lawn, lawn perimeter, and the house wall above grass) and 1 product for the 2 surfaces (driveway and garage door and adjacent walls) receiving different application practices (Table 1). Different active ingredients had to be chosen for each of the 5 surfaces in both sets of plots (historic and revised treatment practices) in order to determine the source of pyrethroids in the runoff water. Contributing to the selection of the products were the preliminary results from a formulation wash-off study conducted on concrete slabs (Harbourt et al., unpublished manuscript; preliminary results are presented in the Supplemental Data), which included tests on 17 products. The product chosen for the driveway was a product included in all Pyrethroid Working Group experiments. The 2 products chosen for the lawn treatments were the products with the 2 highest sales for lawn applications in California. Products with contrasting wash-off behavior on concrete were chosen for the lawn perimeter and house wall next to grass. The only product not registered for residential use was Warrior®, and its application to residential lawns was similar to its agricultural uses.

**Table 1:** Products applied to each of the test surfaces

Surface	Historic practices treatment (lots 1, 3, and 5)	Revised practices treatment (lots 2, 4, and 6)
Lawn	DeltaGuard G (deltamethrin)	Talstar PL granular (bifenthrin)
Grass perimeter	Demand CS (λ-cyhalothrin)	Warrier (λ-cyhalothrin)
House wall	Wisdom TC (bifenthrin)	Prelude (permethrin)
Garage door	Tempo Ultra SC (β-cyfluthrin)	Tempo Ultra SC (β-cyfluthrin)
Driveway	Cynoff WP (cypermethrin)	Cynoff WP (cypermethrin)

### Product application procedures

Calibrated applications of all 5 products were made on 2 August 2011 to all 6 house lots and repeated (except for the broadcast application to the lawn) on 4 October, 6 December, 2 February, 3 April, and 5 June. All application rates were at the maximum label rate for the given product and specific concentration.

#### Lawn

Applications of the lawn products were made using a drop spreader, and any material landing on the driveway or street curb was swept back onto the lawn.

#### Grass perimeter

The pyrethroid was applied to the grass in a band 1.5 m wide, measured from the wall outward.

#### Wall above grass

The pyrethroid was applied to the vertical wall above the grass in a band measuring 0.61 m high. The application stopped approximately 10 cm (horizontally) from the section of wall above the concrete driveway to prevent wash-off water containing pyrethroids from this surface from running down the driveway.

#### Garage door and wall above driveway

The pyrethroid was applied differently for the historic and revised application practices. For both practices, it was applied to the wall directly above the concrete driveway in a band 0.61 m high, starting at the surface of the driveway. In the treatment representing use according to historic application practices, the pyrethroid was also applied to the garage door in a band 0.61 m high starting at the surface of the driveway. In the treatment representing the revised application practices, the garage door was not treated.

#### Driveway

The pyrethroid was applied differently for the historic and revised application practices. Historic practices had pyrethroid applied to all of the upper part of the driveway in a band 1.5 m wide, beginning at the wall or garage door. Revised practices treated only the expansion joint between the garage door and the driveway.

### Irrigation and simulated rainfall and amounts

The lawns of the 6 house lots were managed by a local lawn service, including mowing and setting the irrigation schedule. The irrigation schedule was adjusted to match both the duration and the number of days per week for a typical lawn in central California. Typically, an irrigation event lasted anywhere from 8 min to 15 min and applied from 3.4 mm to 6.4 mm of water, depending on the needs for maintaining the health of the lawn, with some spray reaching the driveway in a pattern that depended on the wind.

A rainfall simulator was used to supplement natural rainfall and to produce storm events representative of Sacramento (CA, USA) during the period of October to March. The rainfall intensity was set at approximately 12.7 mm per hour, with the duration of each event varying to meet the desired rainfall amount. Using a 15-yr rainfall record for Sacramento, 1-in-5-yr and 1-in-2-yr rainfall events were determined. The 1-in-5-yr rainfall event in October through March varied from 9.7 mm to 22 mm, and the 1-in-2-yr rainfall event ranged from 9.7 mm to 16 mm in November to March. The intent was to have a 1-in-5-yr event in October through March and an additional 1-in-2-yr event in November through March, with either natural or simulated rainfall. This was accomplished with the following algorithm: In October, a simulated 1-in-5-yr event would be conducted in the second week if a natural event of the same magnitude had not previously occurred. In November through March, a simulated rainfall event would be conducted unless at least a 1-in-2-yr natural rainfall event had occurred. If at least a 1-in-2-yr natural rainfall event had not occurred, then the simulated event would be a 1-in-5-yr storm. In the fourth week of the month, if a 1-in-5-yr rainfall event (simulated or natural) had not occurred, then a simulated 1-in-5-yr rainfall event would be performed. If a 1-in-5-yr rainfall event (simulated or natural) had occurred, but an additional event equal to or greater than a 1-in-2-yr rainfall event had not occurred during the month, then a simulated 1-in-2-yr event would be performed.

### Runoff sampling and monitoring

The runoff volume from each house lot was measured and recorded at the collection point down-gradient from the lot. Runoff was defined as the water leaving the house lot and entering the collection device. A 38-L stainless-steel collection basin contained a tipping bucket apparatus for capturing both the flow rate and runoff volume. The tipping bucket was constructed of stainless steel, designed and calibrated to collect approximately 100 mL to 200 mL of water before tipping (exact amount was determined for each collection basin). When the tipping bucket tipped, a signal was recorded by a data-logging system along with the time at which the tip occurred. The number of tips was then counted to determine the volume of water collected as well as the rate of flow over time. When very large runoff events occurred, the tipping bucket was unable to record the number of tips accurately for determination of the flow rate. In such a case, a sump pump located in the 38-L stainless steel basin was used to measure the flow rate by logging the start and stop times of the pump and calculating the volume of flow transferred by the pump based on verification runs performed during installation (and periodically during maintenance).

Event-based sampling was performed for irrigation and rainfall (natural or simulated) events, with a refrigerated autosampler triggered to collect water samples when a runoff event occurred through an autosampler intake in the stainless steel collection basin after a predetermined number of tips (depending on each individual tipping bucket calibration). Approximately 1 composite sample was collected for each lawn irrigation event into a 1-L glass bottle. When a rainfall (either natural or simulated) runoff event occurred, a series of up to 12 composite samples was collected into 1-L glass bottles. Samples were refrigerated during collection and remained refrigerated until study personnel retrieved samples from the autosamplers, at which time samples were immediately capped with Teflon-lined lids. Sample lids were secured with electrical tape prior to sample storage. Samples remained refrigerated from the time of collection until they were shipped to the analytical laboratory. Approximately 350 µL formic acid (10% solution) was added to each sample collection bottle prior to collection of water in order to maintain the pH below 6. On collection of the runoff sample, approximately 50 mL methanol was added to reduce sorptive loss to the glass during storage and aid in the extraction of the test substances. All samples were stored cold to minimize degradation during storage and shipment to the analytical laboratory. Samples were transported on cold/blue ice directly to the analytical laboratory by research personnel.

Because of the range of precipitation amounts and intensities produced by lawn irrigation events, natural rainfall events, and simulated rainfall events, autosampler sampling programs were developed and tailored to the type of precipitation event occurring and also to account for the variation in runoff volumes throughout the year.

### Analysis

All sample analyses were performed at Morse Laboratories. Residues of bifenthrin, cypermethrin, beta-cyfluthrin, deltamethrin, lambda-cyhalothrin, and permethrin were extracted from water samples by first adding methanol and sodium chloride to each sample, then partitioning the mixture twice with hexane. The upper hexane layer was passed through sodium sulfate, evaporated, and brought to a known volume of hexane. An aliquot of the hexane extract was then subjected to Bond Elut™ LRC-Si solid-phase extraction (SPE), eluted with hexane:diethyl ether (9:1, v/v), evaporated to dryness, and reconstituted with a 10/20/100-ng/mL internal standard solution. Determination of pyrethroid residues was conducted using gas chromatography with mass selective detection using negative chemical ionization (GC–MSD/NCI). The targeted (method) limits of quantitation for residues in water samples were 2.0 ng/L for bifenthrin, cypermethrin, beta-cyfluthrin, and lambda-cyhalothrin; 4.0 ng/L for deltamethrin; and 20.0 ng/L for permethrin. Chromatography conditions are provided in the Supplemental Data.

Samples were analyzed in groups or sets, consisting of the number of runoff samples that can effectively be managed through the analytical procedure at one time, plus at least 1 control sample and at least 1 fortified recovery sample. Fortification levels ranged from limits of quantitation to a level that encompasses the highest residues found. Select runoff samples, blind spikes, field blanks and duplicates, and field spikes were analyzed. Because of the high volume of runoff samples, it was neither feasible nor necessary to analyze all samples for the duration of the study. However, all samples collected in the 2 wk following an application, as well as all samples collected during large rainfall events, were analyzed. If significant residues were still present beyond the 2-wk period after application, additional samples were analyzed. Typically, an individual sample concentration greater than 100 ng/L from any house lot prompted the analysis of additional runoff samples (from all house lots to maintain a consistent comparison). However, no definitive criteria were developed, and what seemed significant might have changed with time during the study.

Approximately 10% of the total number of runoff samples was submitted as field blanks, blind spikes, or field duplicates; approximately 5% of the total number of runoff samples was submitted as field blanks to determine whether interferences were introduced, and approximately 5% of the total samples consisted of blind spikes to measure accuracy. Triplicate blind spikes were prepared and analyzed to measure analytical precision. Field blank samples were collected and exposed to the site conditions and preservation techniques similar to runoff samples from collection through shipment to the laboratory. The source water for the field blanks was the groundwater source used for lawn irrigation and simulated rainfall. The field spiking levels for the transit stability studies were approximately 0 ×, 10 ×, 40 ×, and 100× the limits of quantitation. Fortified samples were prepared in triplicate using the same preservation procedures as for runoff samples. The fortified samples were submitted to the laboratory as blind field spikes. Approximately 5% of the total sample count was field spikes. Results of these quality-control samples are presented in the Supplemental Data.

## Results and Discussion

From the time of the first application on 2 August 2011 through the end of the study on 1 August 2012, 34 rainfall events (simulated and natural) occurred, totaling approximately 318 mm rainfall. Eight simulated events totaling approximately 146 mm and 26 natural events totaling approximately 171 mm occurred. During the same period, Sacramento experienced approximately 48 rainfall events totaling approximately 343 mm rainfall. Also during this time, 185 lawn irrigation events occurred. In total, 1709 runoff samples were collected, and 1149 of these samples were analyzed. Some samples, primarily from lawn irrigation events, were not analyzed because residues detected in sample analyses had declined considerably in runoff samples analyzed since the latest application, so these events would not significantly contribute to the runoff losses that had already occurred. This section presents and discusses the relative runoff losses from the different surfaces, followed by a discussion of the results for each of the 5 surfaces.

### Relative runoff losses from the different surfaces

**Figure 2:**
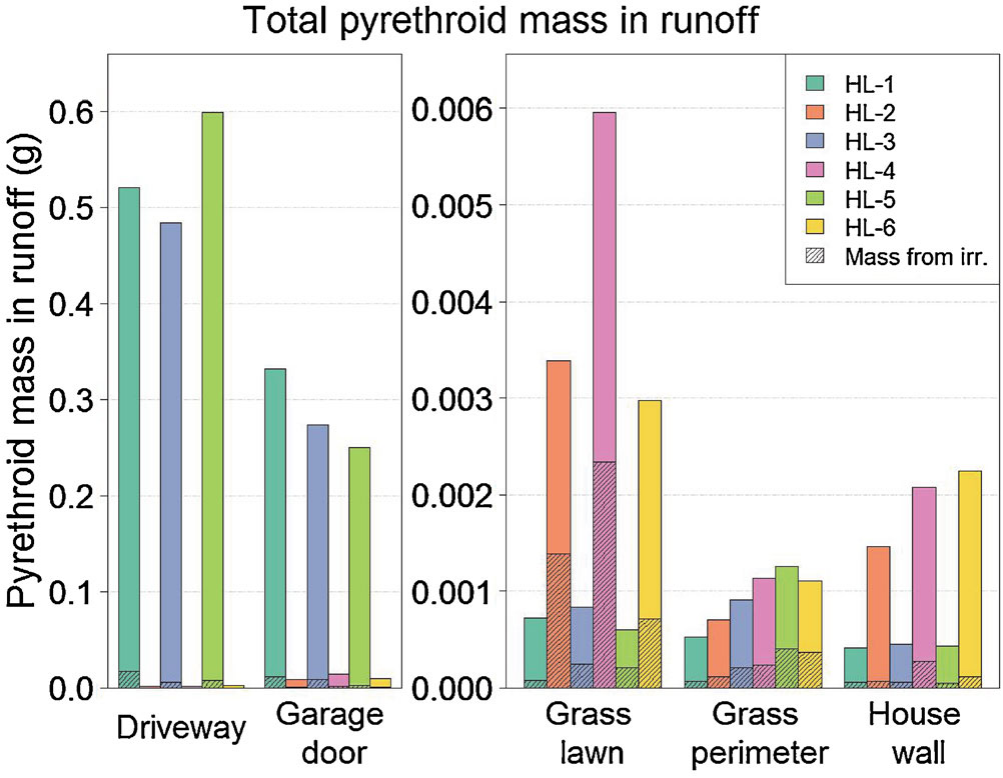
Total measured pyrethroid mass in runoff from each surface and for all 6 application periods. Note that the *y* axis scale is different in the 2 graphs for visual clarity. HL = house lot.

**Figure 3:**
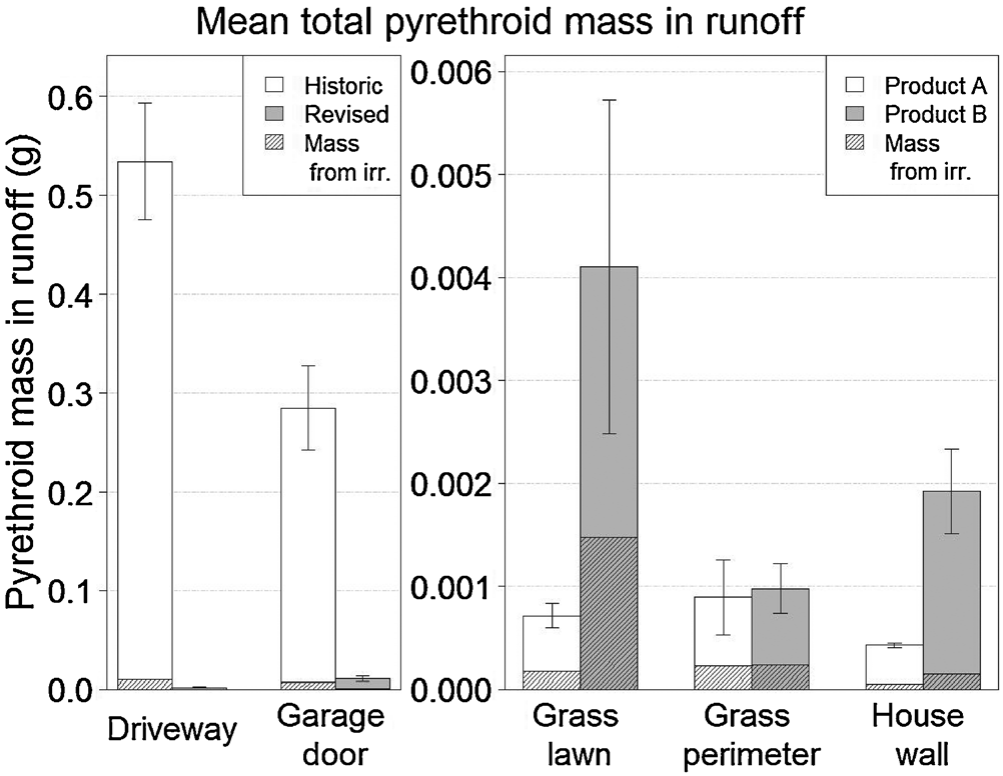
Total measured pyrethroid mass in runoff for all 6 application periods expressed as an average of the 3 replicate house lots. Note that the *y* axis scale is different in the 2 graphs for visual clarity. Product A and product B denote the difference in product applied to the grass lawn, grass perimeter, and house wall surfaces; the application practice was the same for all house lots. Error bars represent 1 standard deviation from the mean.

**Figure 4:**
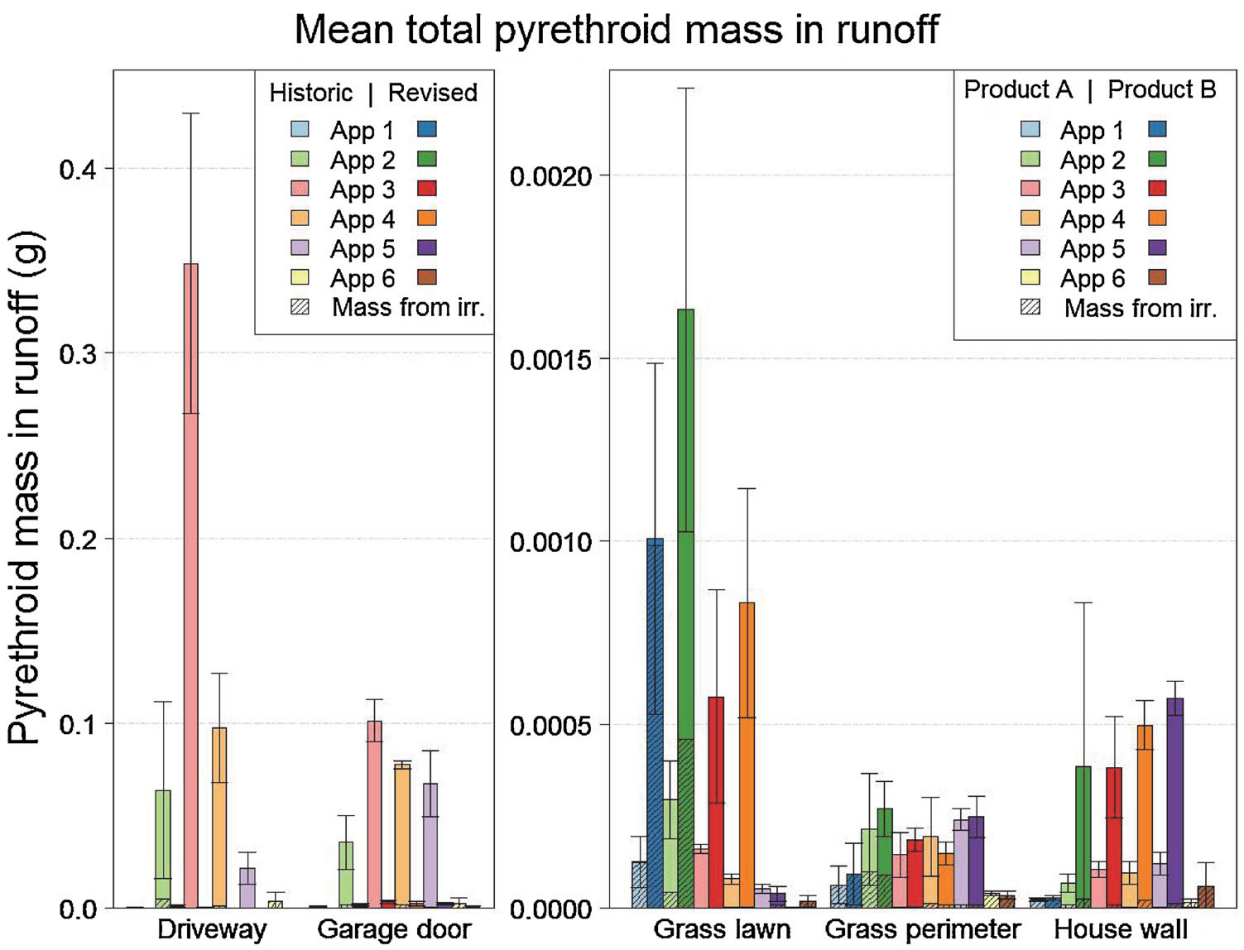
Total measured pyrethroid mass in runoff following each application (App) event expressed as an average of the 3 replicate house lots. Note that the *y* axis scale is different in the 2 graphs for visual clarity. Product A and product B denote the difference in product applied to the grass lawn, grass perimeter, and house wall surfaces; the application practice was the same for all house lots. Error bars represent 1 standard deviation from the mean.

Figure 2 shows the runoff losses from each of the surfaces for each of the 6 house lots (house lots 1, 3, and 5 represent the historic application practices for the driveway and garage areas; house lots 2, 4, and 6 represent the revised application practices for the driveway and garage areas). Figure 3 presents the same data as an average of the 3 replicate plots. These figures show that most of the runoff losses that occurred with the historic application practices occurred from the driveway. However, the amount of runoff losses was reduced dramatically from both the driveway and the garage using the revised application practices. Overall runoff losses were more than 40 times lower from the house lots with the revised practices compared with the house lots with the historic practices as a result of the lower amounts applied in the revised practice treatments as well as a lower percentage of material applied running off. Figure 4 shows a breakdown of the losses in each of the 2-mo periods following the 6 applications. The driveway and the garage area were the major sources of runoff losses in each of the 6 periods and in each period were reduced significantly by the revised application procedures.

Losses expressed as percentages of the total losses are shown in Table 2, further illustrating that nearly all of the runoff losses occurred from the applications to driveway and the garage door area when using the historic application practices. This is slightly different in the intervals between the first and second applications, where the other 3 surfaces contributed 13% of the material present in the runoff water. The higher contribution of the lawn, grass perimeter, and house wall is not surprising because much of the driveway and garage door area received minimal drift from the lawn sprinkler system. In addition, essentially no rainfall occurred in the first 2 mo of the study, which helps to explain why runoff losses were quite low compared with the rest of the study. Also, the grass lawn products were applied to the lawn only once, during the first application event on 2 August 2011. Thus, for both application strategies, the amount of lawn-applied chemical found in the runoff tends to decrease after the first 2-mo monitoring period. As previously mentioned, overall runoff losses from the house lots with the revised application practices were lower by a factor of approximately 40, with essentially all of this reduction coming from the driveway and garage door surfaces. As a result of the significant decrease in runoff losses from the driveway and the garage walls, the other 3 surfaces became more important contributors to overall runoff losses with the revised application practices.

**Table 2:** Runoff losses from the individual surfaces as a percentage of total measured runoff losses

Interval	Runoff losses from the specific surface (% of total runoff losses)				
	Driveway	Garage wall	Grass lawn	Grass perimeter	House wall
Historic practices (lots 1, 3, and 5)					
Application 1 to study end	65	35	0.087	0.11	0.052
Applications 1 to 2	27	60	7.7	3.8	1.5
Applications 2 to 3	64	36	0.30	0.22	0.069
Applications 3 to 4	77	22	0.036	0.032	0.024
Applications 4 to 5	56	44	0.046	0.11	0.055
Applications 5 to 6	24	75	0.058	0.27	0.14
Application 6 to study end	55	44	0.035	0.66	0.22
Revised practices (lots 2, 4, and 6)					
Application 1 to study end	10	54	21	5.0	9.8
Applications 1 to 2	0.36	11	79	7.3	2.2
Applications 2 to 3	25	34	30	4.9	7.0
Applications 3 to 4	7.8	70	11	3.7	7.6
Applications 4 to 5	4.0	58	22	3.9	13
Applications 5 to 6	2.0	73	1.1	7.1	16
Application 6 to study end	0.52	79	3.3	6.3	11

**Figure 5:**
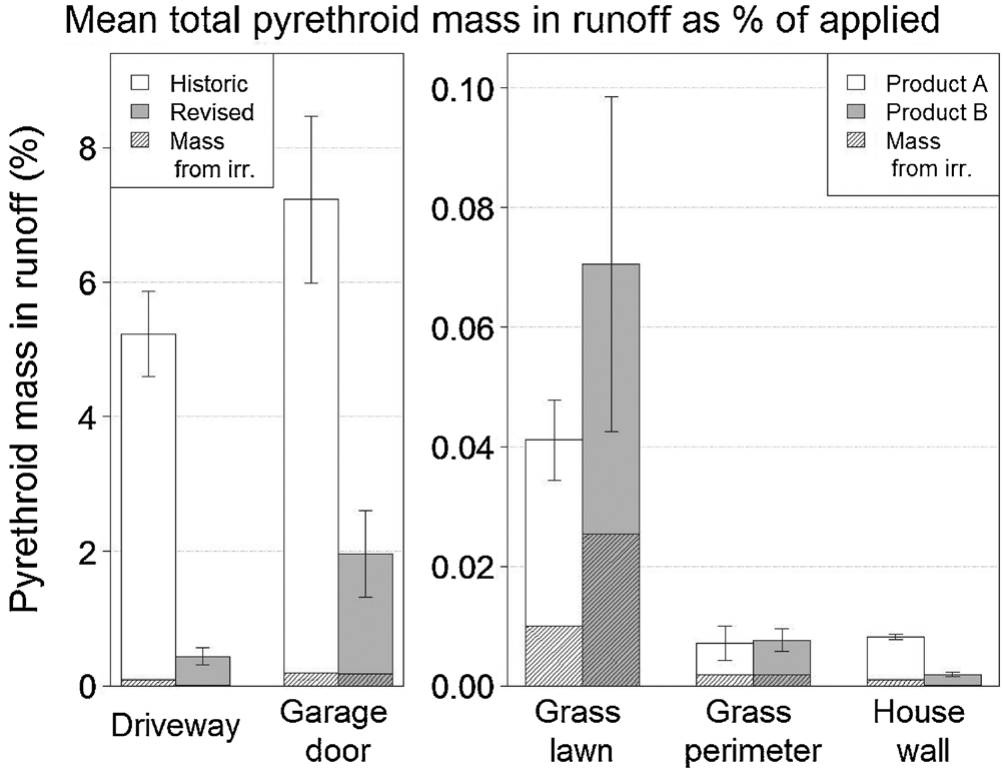
Total measured pyrethroid mass expressed as a percentage of chemical applied in runoff for the duration of the study expressed as an average for the 3 replicate house plots. Note that the *y* axis scale is different in the 2 graphs for visual clarity. Error bars represent 1 standard deviation from the mean.

Figure 5 shows the runoff losses from all 5 surfaces presented as a percentage of the applied amount rather than as total mass as in Figures 2 and 3. Runoff losses expressed as a percentage of chemical applied are highest for the driveway and garage door surfaces, regardless of whether the applications were made according to the historic or revised practices. However, runoff losses expressed as a percentage of chemical applied are lower with the revised application practices for the driveway and garage door surfaces. Also, note that most of the runoff losses occurred as a result of rainfall rather than irrigation. The differences between wash-off expressed as mass and percentage of applied in runoff are discussed below for each of the 5 application surfaces.

The results of the present study are specific to the choice of products and their associated properties that might affect wash-off. However, recent work [11] has shown that large differences between formulations in small-scale experiments were much smaller (and sometimes in the opposite direction) under actual-scale use conditions. Therefore, the general conclusions that the main source of residues under the historic practices will be the driveway and associated vertical surfaces and that revised application procedures will significantly reduce residues compared with historic applications are generally valid, although the differences will vary depending on the specific products.

### Driveway

The driveway was the largest contributor of pyrethroids for historic application practices but not with the revised application practices. Approximately 30 times more material was applied to the driveway with the historic practices than with the revised application, and the runoff losses as a percentage of chemical applied were, on average, a factor of 12 less with the revised application practices, presumably because of the placement of the material into only the expansion joint. As a result, 265 times more pyrethroid mass was recovered in runoff water from the lots with the historic practices, showing that the revised application practices (spot treatment) are an effective management practice for reducing pyrethroid loss in runoff water. Table 3 provides the runoff losses both as mass and as a percentage of applied for the entire study. Note that the application for the revised application practices was 4 times greater (second application event only) than intended. Therefore, the mass losses for the interval between the second and third applications would have presumably been 4 times lower if the correct amount had been applied (assuming no change in the amount of wash-off expressed as a percentage of applied). In the house lots receiving the historic application practices, approximately 66% of the runoff losses occurred between the third and fourth sets of applications, mostly from a 1-in-5-yr simulated rainfall event 2 d after the third set of applications. Four rainfall events, the first significant rainfall event after each respective application, contributed most to the runoff losses from the driveway surface for the historic application practices; these events occurred on 8 December 2011 (application on 6 December), 5 October (application on 4 October), 15 February (application on 2 February), and 11 April (application on 3 April). The ratio of runoff losses from house lots with the historic application practices to those with the revised practices varied with time. For example, the runoff losses expressed as a percentage of chemical applied for the revised application procedures (to correct for the different application rates) were about equal in the periods between the second and third sets of applications and between the third and fourth applications, but the runoff losses with the historic application procedures were a factor of 5 higher in the period between the third and fourth sets of applications compared with the period between the second and third sets of applications. The reason for these differences is related to rainfall amounts and timing and perhaps temperatures and small variations in application practices.

**Table 3:** Comparison of the measured runoff losses from the driveway expressed as a percentage of applied and total measured mass for the entire duration of the study, as well as for each application interval

	Runoff losses from the driveway					
	Percentage of applied			Mass (g)		
Interval	Historic	Revised	Ratio	Historic	Revised	Ratio
Application 1 to study end	5.2	0.43	12	0.53	0.0020	265
Applications 1 to 2	0.026	0.0090	2.9	0.00044	0.0000045	98
Applications 2 to 3	3.6	0.67	5.4	0.064	0.0014	46
Applications 3 to 4	20	0.76	26	0.35	0.00039	900
Applications 4 to 5	5.6	0.29	19	0.097	0.00015	650
Applications 5 to 6	1.3	0.14	9.3	0.021	0.000070	300
Application 6 to study end	0.20	0.00567	36	0.0034	0.0000029	1200

### Garage door and adjacent walls

The garage door and adjacent walls were the second largest source of pyrethroids with historic application practices but were the largest with the revised application practices (Table 2). Table 4 provides the runoff losses both as mass and percentage of applied for the entire duration of the study. The house lots receiving the revised application practices received approximately 7 times less material. Because these same lots contained 25 times less pyrethroid mass in the runoff water, the revised application procedures were an effective management practice for reducing pyrethroid mass in runoff water from the garage door and adjacent walls, as was the case with the driveway surface. Note that the amount applied in the revised applications was somewhat variable because of difficulty in applying to the narrow, 1-foot sections of wall on each side of the garage door. However, the runoff losses as a percentage of applied were also generally less with the revised application practices, likely because the application area did not include the aluminum garage door surface. The report by Trask et al. [5] indicates that wash-off losses are less from stucco than from aluminum. Two exceptions seen in the present study were the periods between the first and second applications and also between the sixth application and the end of the study, in which no natural or simulated rainfall events occurred and drift from the lawn irrigation sprinklers onto the garage door was negligible. Similar to what was found for the driveway, almost 36% of the runoff losses from the house lots receiving the historic application practices occurred between the third and fourth sets of applications; but in this case the runoff, losses were split between 2 events instead of occurring in 1 event. In general, runoff losses from the garage door and adjacent walls did not drop off as quickly following the application as they did from the driveway for house lots with the historic application practices.

**Table 4:** Comparison of the measured runoff losses from the garage door and adjacent walls expressed as a percentage of chemical applied and total measured mass for the entire study duration, as well as each application interval

	Runoff losses from the garage door and adjacent walls					
	Percentage of applied			Mass (g)		
Interval	Historic	Revised	Ratio	Historic	Revised	Ratio
Application 1 to study end	7.2	1.9	3.8	0.28	0.0110	25
Applications 1 to 2	0.15	0.21	0.71	0.00098	0.00014	7.0
Applications 2 to 3	5.3	1.4	3.8	0.036	0.0019	19
Applications 3 to 4	15	3.3	4.2	0.10	0.0035	29
Applications 4 to 5	12	2.7	4.5	0.077	0.0022	35
Applications 5 to 6	10	3.2	3.1	0.067	0.0025	27
Application 6 to study end	0.42	0.54	0.78	0.0027	0.00044	6.1

### Grass lawn

Overall runoff losses from the lawn were less than 0.1% of overall runoff losses from the house lots with historic application practices (Table 2). There were no differences in application procedures other than what was needed to account for the difference in the granular formulations and application rates, so the differences in results reflect product differences rather than differences in application procedures. The total pyrethroid runoff mass from house lots 1, 3, and 5 was 0.00072 g (0.041% of applied), and the total pyrethroid runoff mass from house lots 2, 4, and 6 was 0.0041 g (0.070% of applied). Wash-off of granules has been proposed as a potential mechanism for runoff of products applied to lawns. However, this does not seem to be the case for these 2 products, because the product applied to house lots 1, 3, and 5 is a gypsum granule, whereas the product applied to house lots 2, 4, and 6 is sand. If wash-off of granules was an important contributing mechanism, the runoff losses of the product applied to house lots 1, 3, and 5 would be expected to be greater. Runoff losses for the individual intervals between the sets of applications are shown in Figure 4.

Note that the only application to the lawn occurred during the first set of applications on 2 August 2011. To produce runoff from the lawn, the soil must be saturated, which occurred for only a portion of the lawn in irrigation and rainfall events. For example, the highest runoff losses in the month of August occurred several days after the amount of lawn irrigation was increased. Therefore, the temporal patterns did not necessarily match the losses from the driveway and garage; however, major rainfall events caused runoff losses from all 5 surfaces, regardless of the time of year. The event with the highest losses occurred between the second and third applications, with an event of similar magnitude occurring during each of the intervals. Presumably, the peak losses from the grass lawn did not occur during December as with the other surfaces because of the length of elapsed time since application, as the lawn was treated only during the first set of applications.

The runoff losses from house lot 4 were somewhat higher than house lots 2 and 6 in many of the larger events throughout the year. The reason for the difference in behavior is not known, but it likely is due to some minor differences among the lots. Runoff in replicate plots in field experiments is often variable, however, and the variation observed among the various plots in the present study is less than is often observed.

The reason for the differences between the 2 products when expressed as a percentage of applied is not clear. The 2 formulations are different, with the product applied on house lots 1, 3, and 5 (the chemical with the lowest losses) being gypsum-based and the product applied on house lots 2, 4, and 6 being sand-based. Given the higher density of the product applied on plots 2, 4, and 6, the potential for carrying product particles in runoff water was higher for the product applied on house lots 1, 3, and 5. If formulation were important, then probably this important factor was the release from the granule. The difference in the Freundlich partitioning coefficient normalized to organic carbon (*K*_OC_) between the 2 active ingredients could also be the cause of the differences in runoff; however, given the difficulty of measuring *K*_OC_ for such strongly bound materials, the current measurements are not adequate for assessing the relative differences in *K*_OC_.

### Grass perimeter runoff losses from the different surfaces

Overall runoff losses from the applications to the grass perimeter lawn were approximately 0.1% of total losses from the house lots receiving historic application practices (Table 2). There were no differences in application procedures, so any differences in results from the 2 sets of house lots reflect product differences rather than differences in application procedures. However, as shown in Table 5, runoff losses were similar for the product applied to house lots 1, 3, and 5 and the product applied to house lots 2, 4, and 6. As indicated in Table 2, the 2 products showed different runoff losses when applied to concrete in a previous study by Harbourt et al. (unpublished manuscript). However, relative wash-off from concrete was not a good predictor of relative runoff in turf for these 2 products. Runoff losses were highest in the interval between applications 5 and 6, although relative losses among intervals were more uniform for the grass perimeter than the other 3 surfaces receiving applications every 2 mo (Figure 4). The pattern of runoff losses does not match the pattern for the other 4 surfaces, even differing from the lawn. However, peak losses tended to occur as a result of major rainfall events and occasionally in response to lawn irrigation events.

**Table 5:** Comparison of the measured runoff losses from the grass perimeter expressed as a percentage of applied and total measured mass for the entire study duration, as well as each application interval

	Runoff losses from the grass perimeter			
	Percentage of applied		Mass (g)	
Interval	Lots 1, 3, and 5	Lots 2, 4, and 6	Lots 1, 3, and 5	Lots 2, 4, and 6
Application 1 to study end	0.0071	0.0076	0.00090	0.0010
Applications 1 to 2	0.0032	0.0046	0.000063	0.000093
Applications 2 to 3	0.0098	0.012	0.00021	0.00027
Applications 3 to 4	0.0068	0.0086	0.00014	0.00019
Applications 4 to 5	0.0090	0.0069	0.00019	0.00015
Applications 5 to 6	0.011	0.011	0.00024	0.00025
Application 6 to study end	0.0019	0.0016	0.000040	0.000035

### House wall

Overall runoff losses from the applications to the house wall were less than 0.1% of total losses from the house lots receiving historic application practices (Table 2). There was no difference in application procedures; thus, differences in results between the 2 sets of house lots reflect product differences, including wash-off characteristics and application rates, rather than differences in application procedures. The product applied to house lots 2, 4, and 6 was applied at a rate approximately 20 times higher than that of the product applied to house lots 1, 3, and 5. The product applied to house lots 1, 3, and 5 had shown greater wash-off losses from concrete expressed as a percentage of applied, as shown in Table 2 from the previous study by Harbourt et al. (unpublished manuscript). As shown in Table 6, overall losses in runoff water expressed as a percentage of applied were about a factor of 5 lower for the product applied to house lots 2, 4, and 6; because of the higher application rate, however, the overall runoff losses expressed in grams were a factor of approximately 4 or 5 higher. The pattern of runoff losses is similar to that observed for the driveways in the house lots with the historic application practices. Five rainfall events contributed most to the runoff losses from driveway for the house lots with the historic application practices: 8 December 2011 (application on 6 December), 5 October (application on 4 October), 15 February (application on 2 February), 11 April (application on 3 April), and 6 June (application on 5 June).

**Table 6:** Comparison of the measured runoff losses from the house wall expressed as a percentage of applied and total measured mass for the entire study duration, as well as each application interval

	Runoff losses from the house wall			
	Percentage of applied		Mass (g)	
Interval	Lots 1, 3, and 5	Lots 2, 4, and 6	Lots 1, 3, and 5	Lots 2, 4, and 6
Application 1 to study end	0.0081	0.0018	0.00043	0.0019
Applications 1 to 2	0.0027	0.00016	0.000024	0.000028
Applications 2 to 3	0.0078	0.0022	0.000069	0.00038
Applications 3 to 4	0.012	0.0021	0.00011	0.00038
Applications 4 to 5	0.011	0.0029	0.000096	0.00050
Applications 5 to 6	0.014	0.0033	0.00012	0.00057
Application 6 to study end	0.0016	0.00034	0.000014	0.000059

## Conclusions

Results from the house lots with the historic application procedures showed that losses of the compounds applied to the driveway and garage door (including the adjacent walls) were 99.75% of total measured runoff losses. The highest losses were associated with significant rainfall events rather than lawn irrigation events. Also, natural and simulated rainfall events accounted for the majority of mass loss from the study site compared with mass loss under lawn irrigation and its associated “urban drool.” Furthermore, runoff losses were 40 times less using the revised application procedures recently specified on pyrethroid labels.

## SUPPLEMENTAL DATA

**Sections S1–S7.** (2.9 MB PDF).

## Supporting Information

All Supplemental Data may be found in the online version of this article.
