# Supplementary material for: MAJOR TRANSPORT MECHANISMS OF PYRETHROIDS IN RESIDENTIAL SETTINGS AND EFFECTS OF MITIGATION MEASURES
Source: Environ Toxicol Chem. 2013 Oct 4;33(1):52–60. doi: 10.1002/etc.2411 (PMC4255737; doi:10.1002/etc.2411)
Supplement: Supplementary file 1 — Supplemental Data [file etc0033-0052-sd1.pdf]

Major Transport Mechanisms of Pyrethroids in Residential Settings and Effects of Mitigation Measures

Paul C. Davidson, Russell L. Jones, Christopher M. Harbourt, Paul Hendley, Gregory E. Goodwin, and

Bradley A. Sliz

Supplemental Material

Table of Contents

S1. Pictures of the Experimental Site 2
S2. Description of the Weather Station 3
S3. Formulation Washoff Study Results 3
S4. Application Equipment and Calibration 4
S5. Analytical Parameters for Water and Tank Mix Samples 6
S6. Summary of the Performance of the Analytical Methold 8
S7. Runoff Losses by Event 11

S1. Pictures of the Experimental Site

Figure S1-1. Individual house lot.

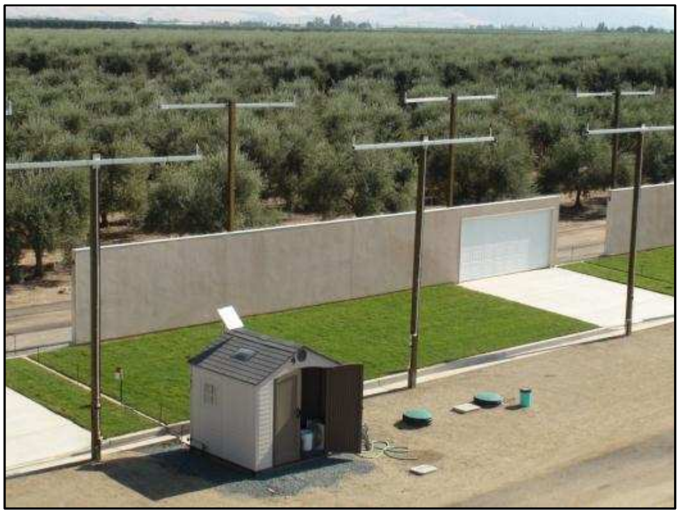

Figure S1-2. Experimental site.

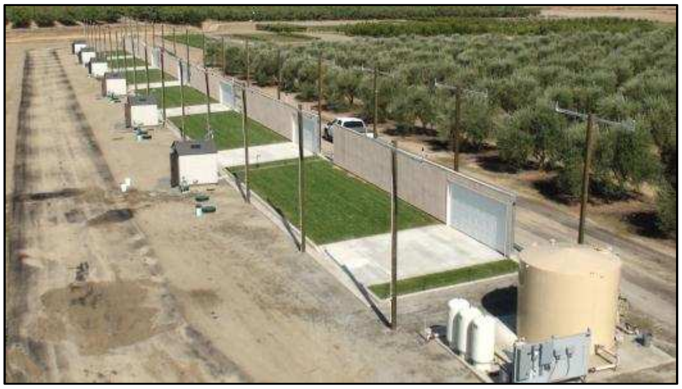

Figure S1-3. Movement from curb to flow and sampling equipment.

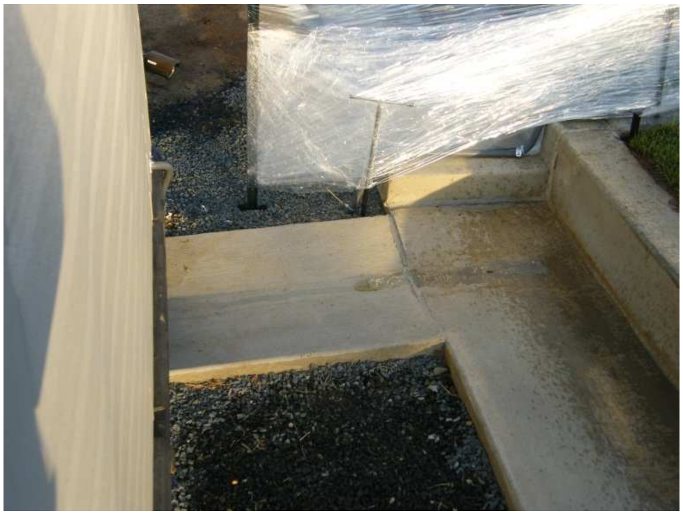

S2. Description of the Weather Station

Over the duration of the study, meteorological data was collected for the sampling site by a weather station. Air temperature, precipitation, relative humidity, wind speed and direction, barometric pressure, and solar radiation were measured. The weather station consisted of a Vaisala WXT520 multi-parameter weather transmitter, a Campbell Scientific CS300 pyranometer, and a Campbell Scientific CR800 datalogger, all of which were mounted to a vertical post. All meteorological measurements were logged every 15 minutes.

S3. Formulation Washoff Study Results

All of the non-granular products were selected from those included in a washoff study by Harbourt et al. (in preparation) in which slabs were subjected to a 1 inch rainfall over a duration of one hour, occurring approximately 24 hours after application. Preliminary washoff results from this study are summarized in Table S3-1.

Table S3-1. Preliminary washoff results from Harbourt et al. washoff study.

Product	Active Ingredient	Washoff (% of applied)[a]
Cynoff® WP	cypermethrin	9.30
Cynoff ®EC	cypermethrin	0.11
Demon® Max	cypermethrin	0.09
Demon® WP	cypermethrin	9.97
Demand® CS	lambda-cyhalothrin	10.85
Scimitar® GC	lambda-cyhalothrin	12.22
Warrior®	lambda-cyhalothrin	0.32
Suspend® SC	deltamethrin	16.81
Dragnet® SFR	permethrin	0.11
Prelude®	permethrin	0.10
Talstar® Professional	bifenthrin	5.68
Wisdom® TC flowable	bifenthrin	13.48
Cy-kick® CS	cyfluthrin	7.91
Danitol® 2.4 EC	fenpropathrin	0.29
Ortho® Bug-B-Gon	esfenvalerate	0.43
Tempo® Ultra SC	beta-cyfluthrin	13.65
Tempo® Ultra WP	beta-cyfluthrin	19.41

[a]Average of three values based on application amounts calculated from petri dish analyses.

S4. Application Equipment and Calibration

The equipment used for each application method was calibrated by measuring the output over time and using a determined pass speed to calculate a target pass time and output.

The granular lawn application was only performed at the first application event in August, 2011. The granular lawn applications were made with Scotts Classic Drop Spreaders (Figure S4-1), producing a 20 inch output swath. A separate spreader was used for each treatment scheme (either lots 1, 3, and 5 or lots 2, 4, and 6). The drop spreaders were calibrated by catching the output test substance in a mounted PVC tray over eleven passes of 52 feet and adjusting the output dial to achieve the desired output. This rate was chosen because it represents an area equal to the large section of lawn at each house lot. Three catches were made per treatment. The collected output of each catch was weighed and the average weight was used along with the area to be treated (1020 sq. ft.) to calculate the target output per lawn. Eleven passes were made to the main lawn section (west of each lot's driveway) and two passes were made to each 5-foot grass strip (east of each lot's driveway). The remaining test substance was weighed back to confirm each application output.

Figure S4-1. Scotts Classic Drop Spreader as the granular product is being dispensed. This spreader was only used on lots 2, 4, and 6. A separate drop spreader was used for the remaining three lots.

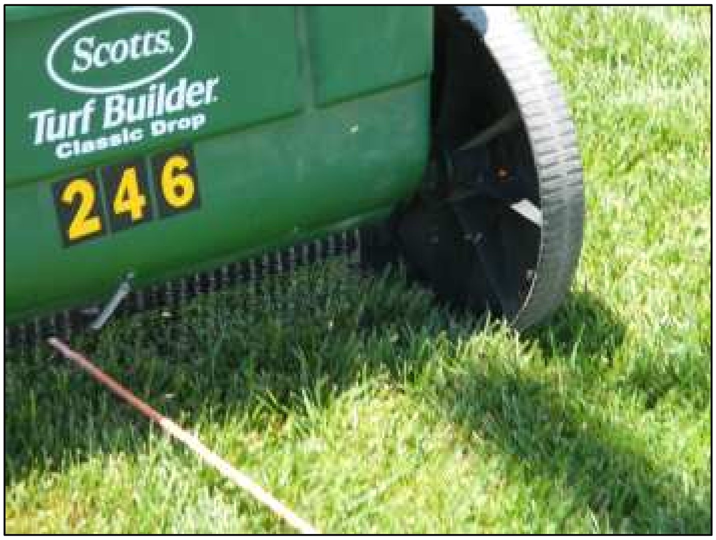

The applications to all remaining surfaces were spray applications. A research grade backpack sprayer (Model GS) was used to complete these applications, with varying spray boom and nozzle arrangements used to provide the desired output rate and distribution. Tank mix volumes and test substance amounts were calculated using the calibrated spray output, plot area, and desired test substance application rate. Target pass times were verified once before each treatment.

Applications to the grass perimeter were made using a three-nozzle boom and Tee Jet flat fan 8010 nozzles. The total treated area for this surface was approximately 285 square feet per plot. Separate spray booms and mix tanks were used for each treatment scheme. A single pass was made on each section of lawn (Figure **Error! Reference source not found.**S4-2).

Figure S4-2. A single pass was made to achieve the grass perimeter application. A three-nozzle spray boom was used to produce the desired spray width of 5 feet.

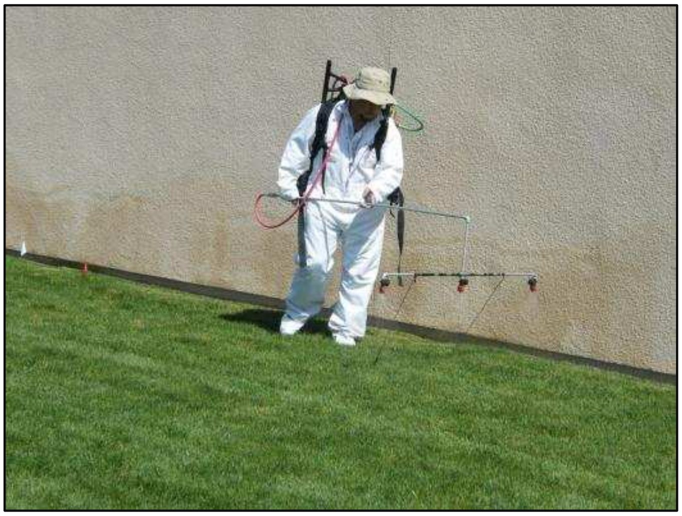

Applications to the house wall were made using a single-nozzle boom and Tee Jet flood jet TK-15 nozzle to treat an area of approximately 93 square feet per plot. Separate booms and mix tanks were used for each treatment scheme. A single pass was made on each wall.

Applications to the garage doors were made using a single-nozzle boom and Tee Jet flood jet TK-15 nozzle to treat an area of approximately 32 square feet per plot. A single pass was made on each garage door.

Applications to the trim beside the garage doors were made using a single-nozzle boom and Tee Jet flat fan 8010 nozzle to treat an area of approximately 2 square feet per plot. Tank mix was pulled from the remaining volume of the garage door applications. A single pass was made on the trim (one-foot section of wall) of each side of the garage door.

Applications to the driveway were made using a three-nozzle boom and Tee Jet flat fan 8008 nozzles to treat an area of approximately 90 square feet per plot. A single pass was made on each driveway. For the revised practices (lots 2, 4, and 6), only the expansion joint between the garage door and driveway was treated. Since only a small amount of chemical mix was required to cover the expansion joint, a pipette was used during the first application event to apply exactly the desired amount. While the pipette method sufficiently satisfied the expansion joint application, subsequent applications used a stainless steel hand pump sprayer to more accurately mimic the equipment used by a pest control specialist.

S5. Analytical Parameters for Water and Tank Mix Samples

- Operating Conditions

Instrument:	Agilent gas chromatograph Model 6890 equipped with an Agilent 7683 autosampler and an Agilent 5973N mass-selective detector operated in the negative chemical ionization (NCI) mode. The system is controlled and data processed by an Agilent G1701CA MS ChemStation software.
Column:	30 m × 0.25-mm i.d. × 0.25 µm film thickness, Varian CP-SIL 8CB-MS
Column Flow:	Initial: 0.9 mL/minute, hold 22.0 minutes<br>Rate: 40 mL/minute<br>Final: 1.5 mL/minute, hold 8.17 minutes
Inlet Liner:	4 mm i.d. gooseneck splitless liner packed with CarboFrit™
Injection Volume:	4 µL
Injection Mode:	Pulsed splitless, 15 psi for 1 minute, purge flow to split vent 50 ml/min. @ 2 minutes
Carrier Gas:	Helium
Detector Reagent Gas:	Methane @ 30%

Temperatures: Column:

	Initial:	80℃ hold 1.00 minute
	Rate 1:	40℃/min. to 180℃
	Rate 2:	5℃/min. to 285℃
	Rate 3:	30℃/min.
	Final:	305℃ hold 5.00 minutes
Detector:	300℃	
Injector:	275℃	

Autotuned: 185, 351, and 449 *m/z*

Ions Monitored (as applicable):

	**Target Ion**	**Qualifier 1**	**Qualifier 2**
Bifenthrin:	m/z = 386	m/z = 387	m/z = 241
Cypermethrin:	m/z = 207	m/z = 209	m/z = 171
Beta-Cyfluthrin:	m/z = 207	m/z = 209	m/z = 171
Deltamethrin:	m/z = 299	m/z = 295	m/z = 297
Lambda-cyhalothrin:	m/z = 205	m/z = 241	m/z = 243
Permethrin:	m/z = 207	m/z = 209	----
Bifenthrin-d_6:	m/z = 392	m/z = 393	m/z = 247
Cypermethrin-d_6:	m/z = 213	m/z = 215	m/z = 177
Cyfluthrin-methyl-d_6:	m/z = 213	m/z = 215	m/z = 177
Deltamethrin-d_6:	m/z = 305	m/z = 301	m/z = 303
Lambda-cyhalothrin-d_6:	m/z = 211	m/z = 247	m/z = 249
Permethrin-d_6:	m/z = 213	m/z = 215	----

Dwell: 50 msec

Retention times (as applicable):

Compound	Peak(s)	Approximate Retention Time (min)
Bifenthrin	1	18.1
Cypermethrin	1<br>2<br>3<br>4	22.8<br>23.0<br>23.1<br>23.2
Beta-Cyfluthrin	1<br>2	22.5<br>22.6
Deltamethrin	1<br>2	25.0<br>25.2
Lambda-cyhalothrin	1<br>2	19.4<br>19.8
Permethrin	1<br>2	21.3<br>21.6
Bifenthrin-d_6	1	17.9
Cypermethrin-d_6	1<br>2<br>3<br>4	23.1<br>23.3<br>23.4<br>23.5
Cyfluthrin-methyl-d_6	1<br>2<br>3<br>4	22.3<br>22.4<br>22.5<br>22.6
Deltamethrin-d_6	1<br>2	25.2<br>25.4
Lambda-cyhalothrin-d_6	1<br>2	19.5<br>19.9
Permethrin-d_6	1<br>2	21.4<br>21.7

S6. Summary of the Performance of the Analytical Methold

Analytical method performance was monitored through concurrent analysis of freshly fortified control samples along with field samples. The overall mean, range of procedural recoveries, and standard deviations are summarized in Table S6-1.

Table S6-1. Summary of concurrent (procedural) recoveries in runoff water samples.

Analyte	Range of Fortification (ng/L)	Sample Size (n)	Range of Recoveries (%)	Overall Mean[a] (%) ± std. dev.
**Bifenthrin**	2.0 - 9,000	252	65 - 158	92 ± 12
**Cypermethrin**	2.0 - 1,300,000	252	69 - 130	94 ± 9.9
**Beta-cyfluthrin**	2.0 - 200,000	252	70 - 115	91 ± 8.8
**Deltamethrin**	4.0 - 18,000	252	61 - 121	89 ± 11
**Lambda-cyhalothrin**	2.0 - 35,000	252	67 - 126	93 ± 11
**Permethrin**	20 - 90,000	252	61 - 121	89 ± 10

[a]Corrected for control contribution, if any.

Residue values were not corrected for procedural recovery results.

Field blind spike (transit stability) results for pyrethroids are summarized in Table S6-2. The limit of quantitation (LOQ) for residues in field blind spike samples was 2.0 ng/L for bifenthrin, cypermethrin, beta-cyfluthrin, and lambda-cyhalothrin; 4.0 ng/L for deltamethrin; and 20.0 ng/L for permethrin.

Table S6-2. Residues in field blind spike samples.

Residues of Pyrethroids from Field Blind Spike Samples			
**Analyte**	**Number (n)**	**Fort Level (ng/L)[a]**	**Range of Assay Results[b] (ng/L) (if applicable)**
**Bifenthrin**	18	N/A	ND - <2.0
	18	20.0	12.3 - 17.2
	18	80.0	53.1 - 70.4
	16	200	122 - 190
**Cypermethrin**	18	N/A	ND - <2.0
	18	20.0	13.3 - 21.8
	18	80.0	53.8 - 78.9
	17	200	124 - 249
**Beta-cyfluthrin**	18	N/A	ND - <2.0
	18	20.0	12.7 - 18.6
	18	80.0	53.5 - 72.3
	17	200	125 - 189
**Deltamethrin**	18	N/A	ND
	18	40.0	22.4 - 49.5
	18	160	97.9 - 194
	17	400	228 - 480
**Lambda-cyhalothrin**	15	N/A	ND - <2.0
	15	20.0	12.2 - 22.4
	18	80.0	42.2 - 82.0
	17	200	120 - 218
**Permethrin**	18	N/A	ND - <20.0
	18	200	126 - 200
	18	800	547 - 781
	17	2000	1270 - 2013

[a]N/A = not applicable.
[b]ND = none detected, no observable chromatographic response.

The limit of quantitation (LOQ) for residues in field blank samples was 2.0 ng/L for bifenthrin, cypermethrin, beta-cyfluthrin, and lambda-cyhalothrin; 4.0 ng/L for deltamethrin; and 20.0 ng/L for permethrin.

No pyrethroid residues >LOQ were found for any field blank samples (untreated control water sample) except for one sample (sample ID PISFDBLK01080411PCD) which contained bifenthrin at 8.03 ng/L, beta-cyfluthrin at 4.63 ng/L, and lambda-cyhalothrin at 31.9 ng/L.

The range of residues found for all field blank samples are summarized in Table S6-3.

Table S6-3. Residues in field blank samples.

Residues from Field Blank Samples		
**Analyte**	**Number (n)**	**Range of Assay Results[a,b] (ng/L)**
**Bifenthrin**	62	ND - 8.03
**Cypermethrin**	62	ND - (1.18)
**Beta-cyfluthrin**	62	ND - 4.63
**Deltamethrin**	62	ND
**Lambda-cyhalothrin**	62	ND - 31.9
**Permethrin**	62	ND

[a]ND = none detected, no observable chromatographic response
[b]Values <LOQ but >LOD are reported in parentheses.

S7. Runoff Losses by Event

Figures S7-1-S7-30 provide runoff losses for each of the five surfaces for each of the individual rainfall and irrigation events.

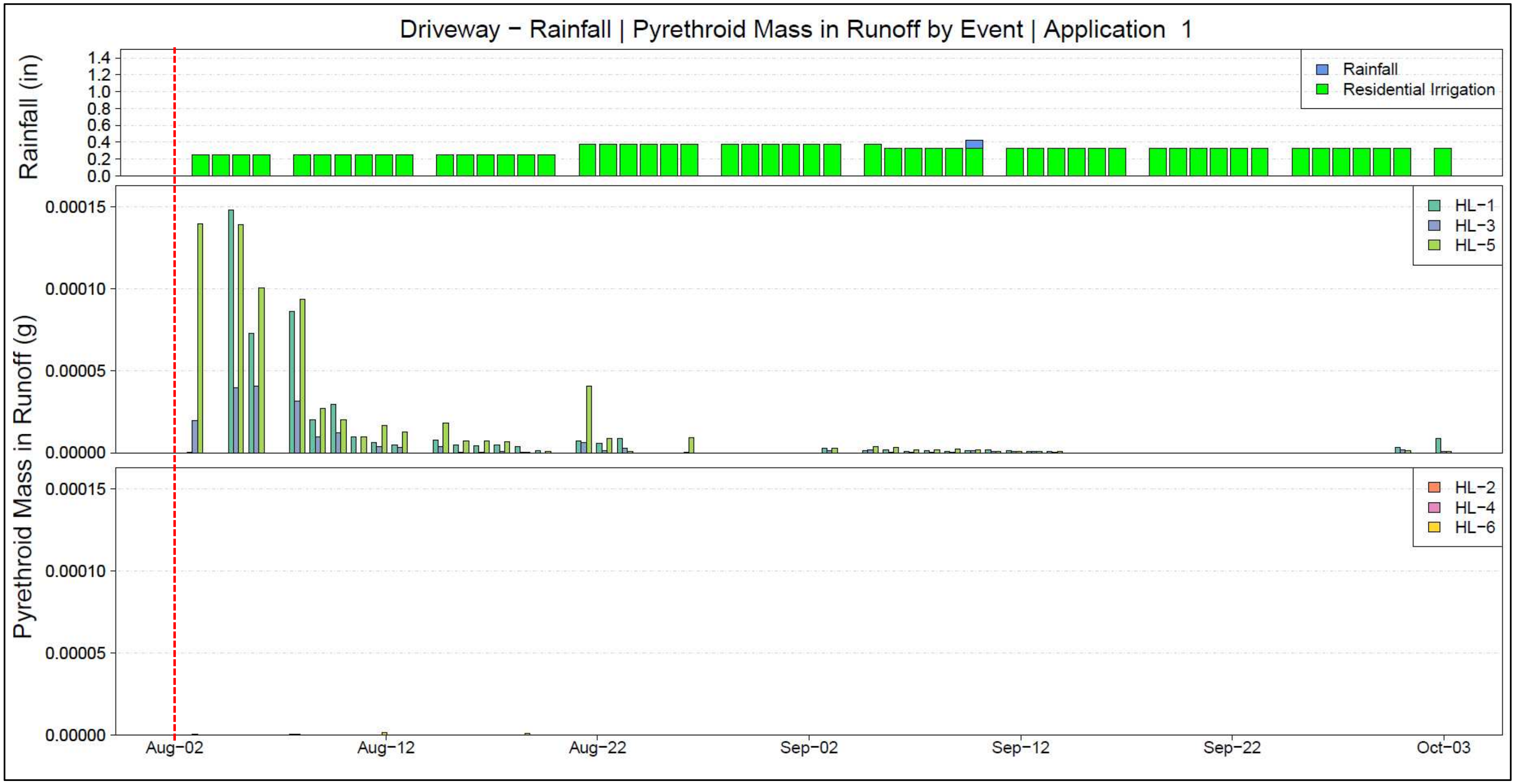

Figure S7-1. Runoff losses from the driveway by event for each of the six house plots between the first and second sets of applications. The first application event occurred on August 2, 2011, as denoted by the vertical red dashed line.

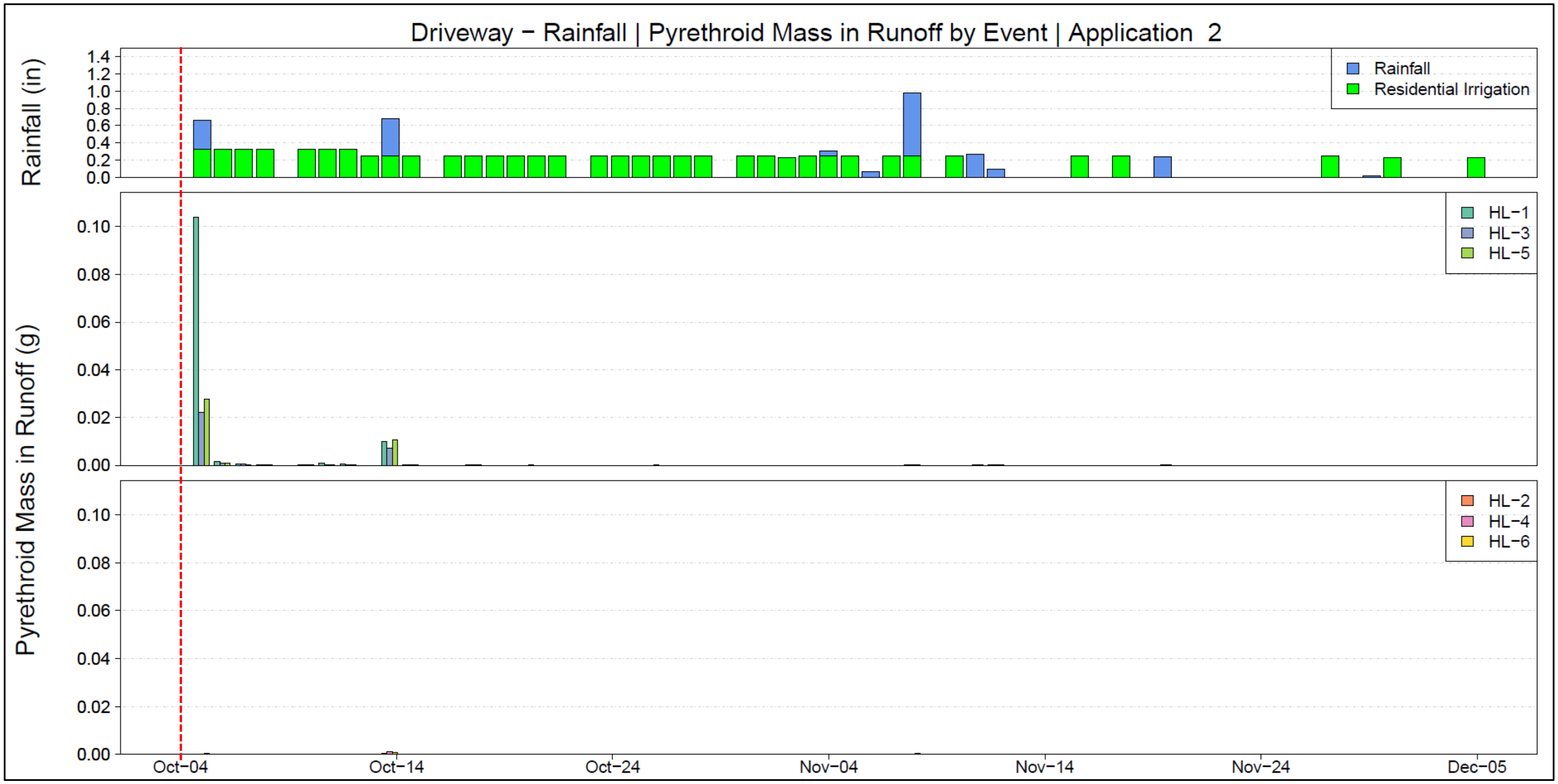

Figure S7-2. Runoff losses from the driveway by event for each of the six house plots between the second and third sets of applications. The second application event occurred on October 4, 2011, as denoted by the vertical red dashed line.

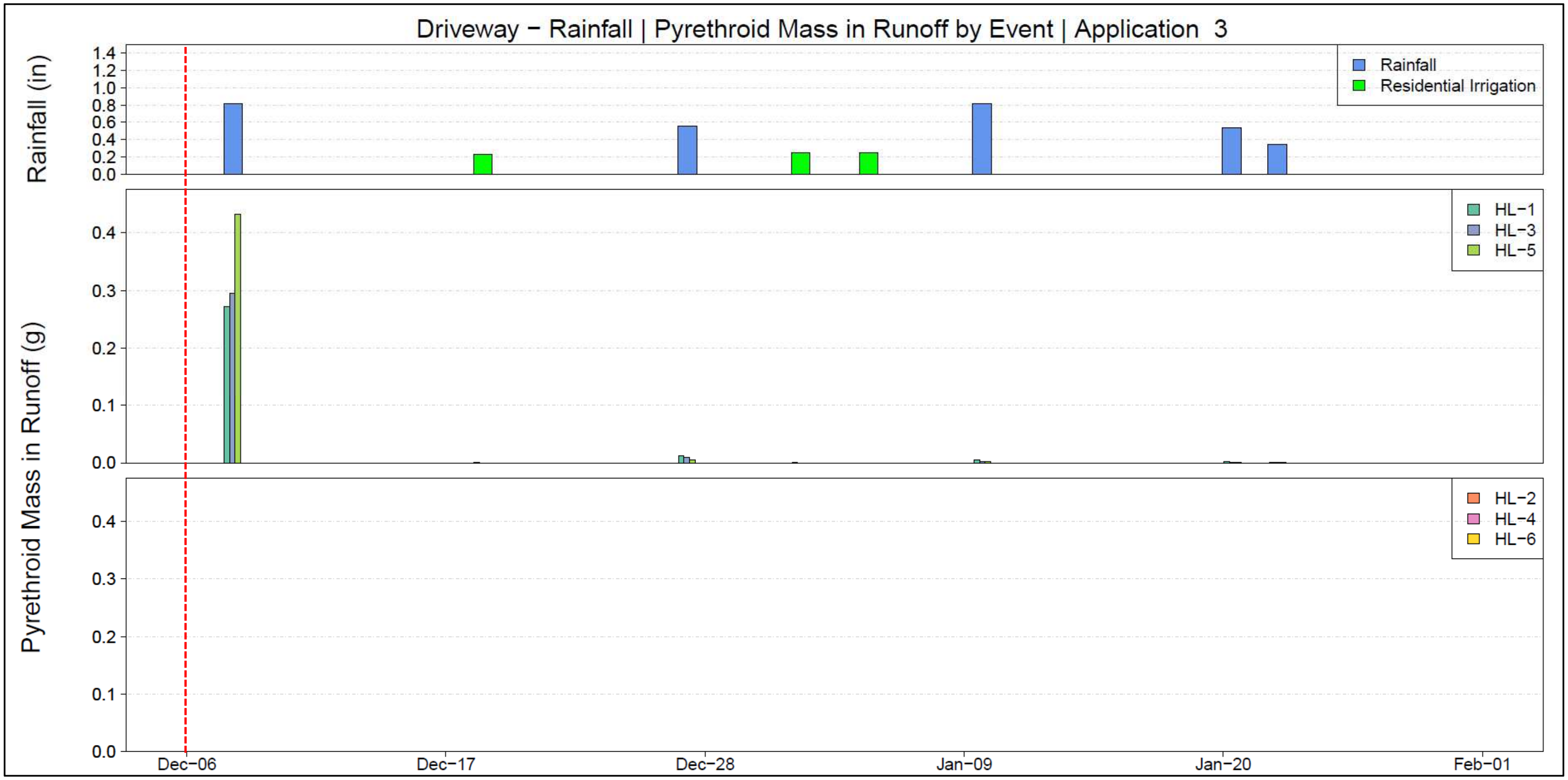

Figure S7-3. Runoff losses from the driveway by event for each of the six house plots between the third and fourth sets of applications. The third application event occurred on December 6, 2011, as denoted by the vertical red dashed line.

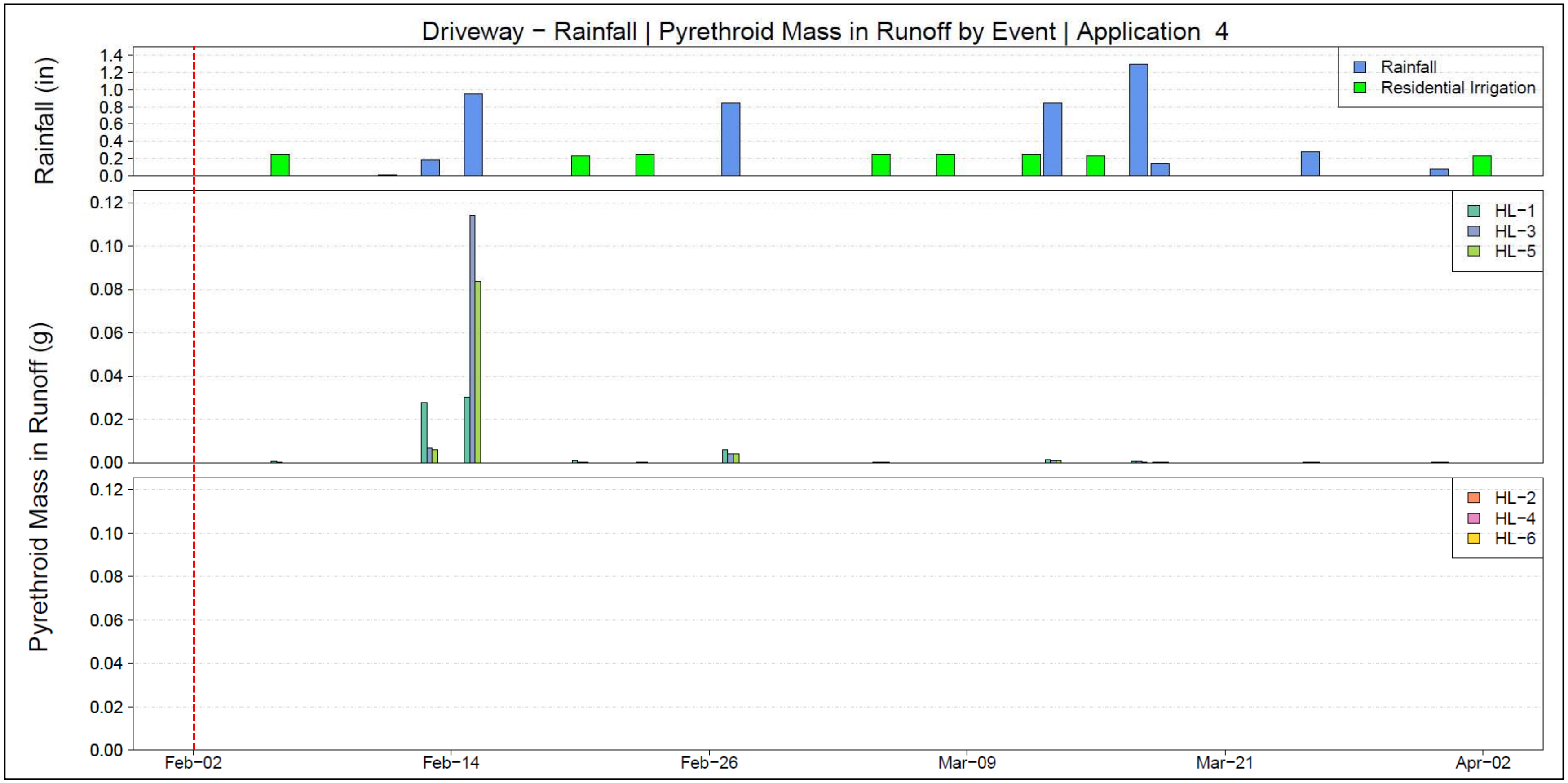

Figure S7-4. Runoff losses from the driveway by event for each of the six house plots between the fourth and fifth sets of applications. The fourth application event occurred on February 2, 2012, as denoted by the vertical red dashed line.

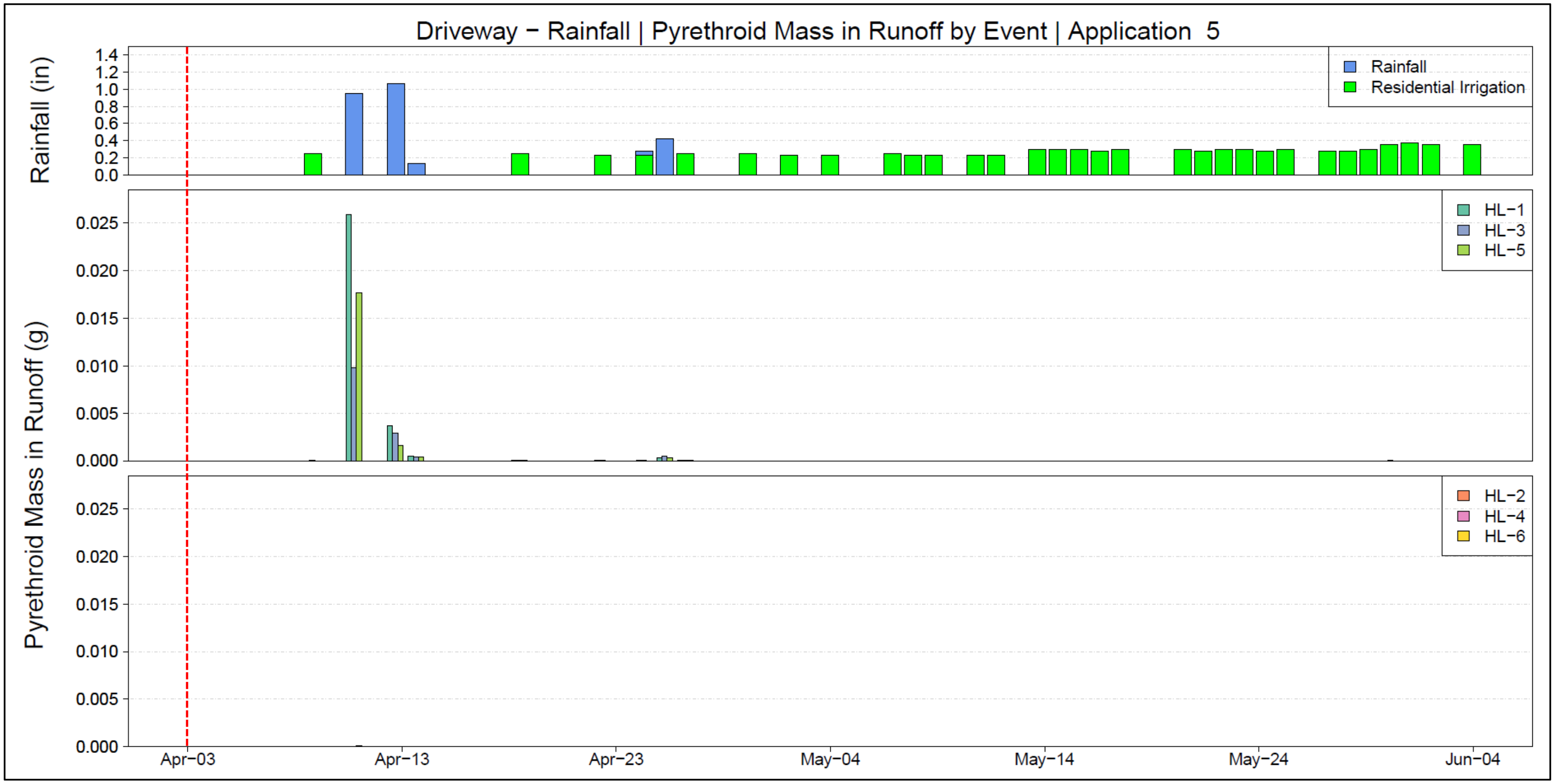

Figure S7-5. Runoff losses from the driveway by event for each of the six house plots between the fifth and sixth sets of applications. The fifth application event occurred on April 3, 2012, as denoted by the vertical red dashed line.

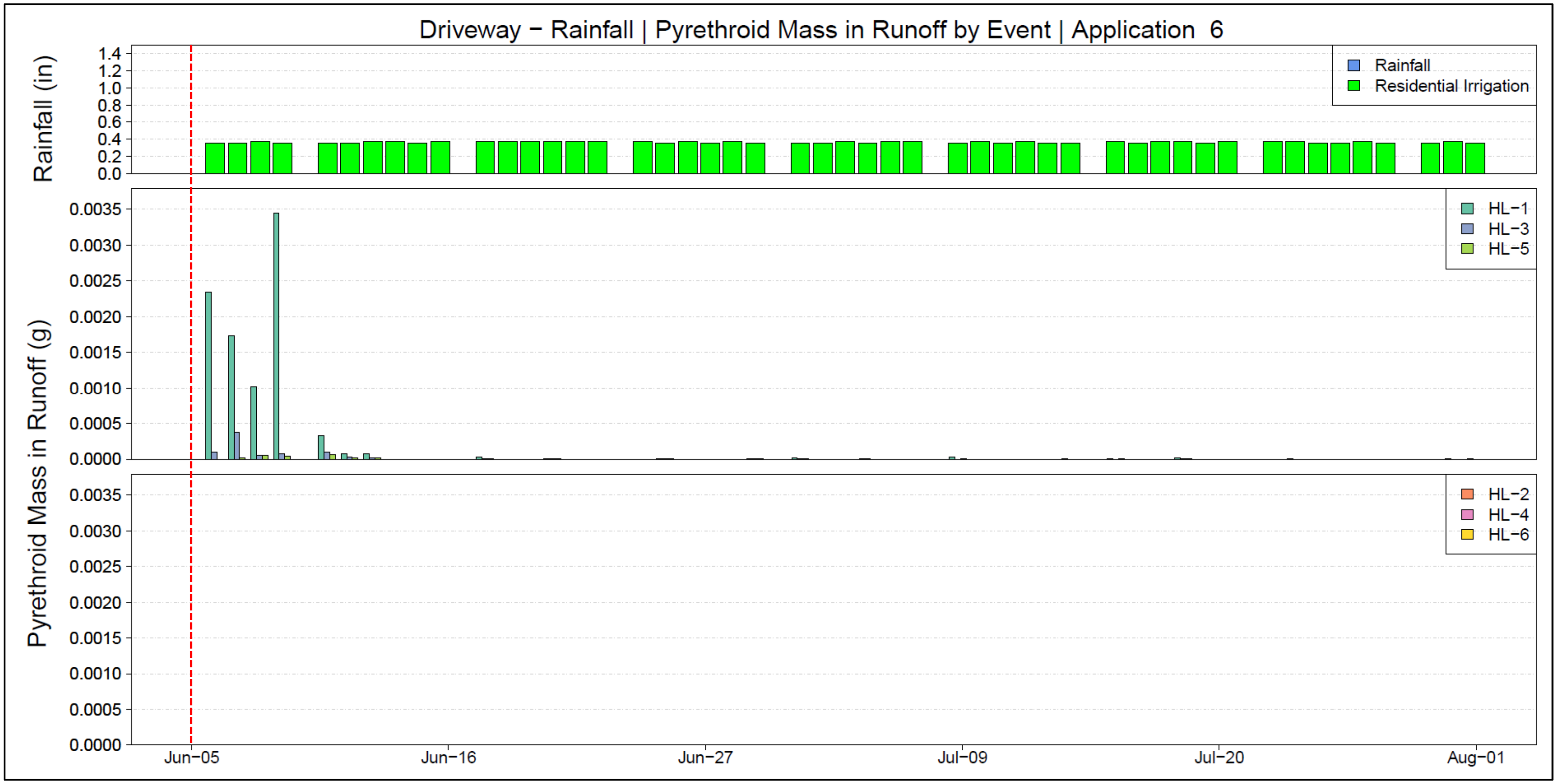

Figure S7-6. Runoff losses from the driveway by event for each of the six house plots between the sixth application and the end of the study. The sixth application event occurred on June 5, 2012, as denoted by the vertical red dashed line.

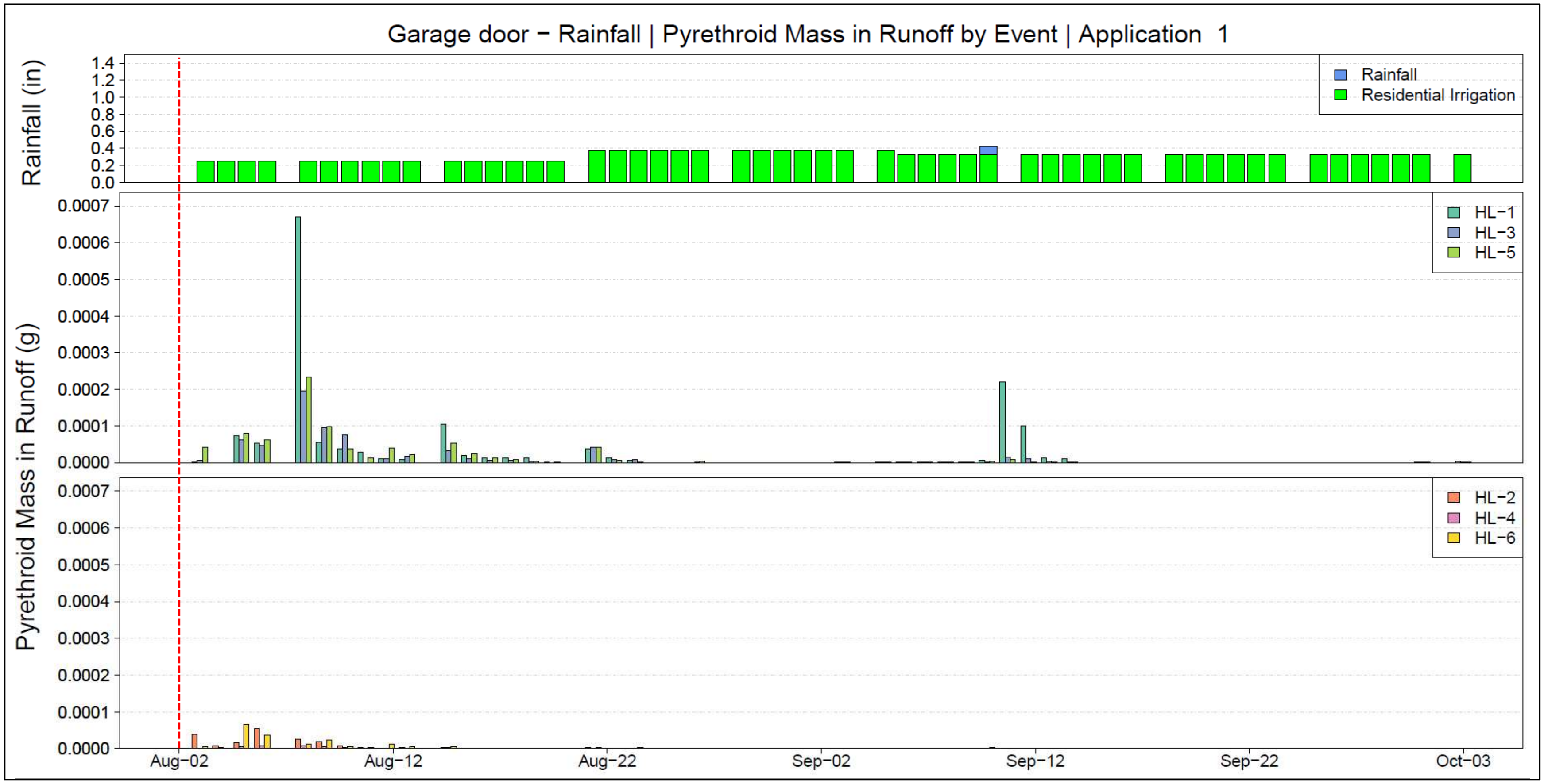

Figure S7-7. Runoff losses from the garage door and adjacent walls by event for each of the six house plots between the first and second sets of applications. The first application event occurred on August 2, 2011, as denoted by the vertical red dashed line.

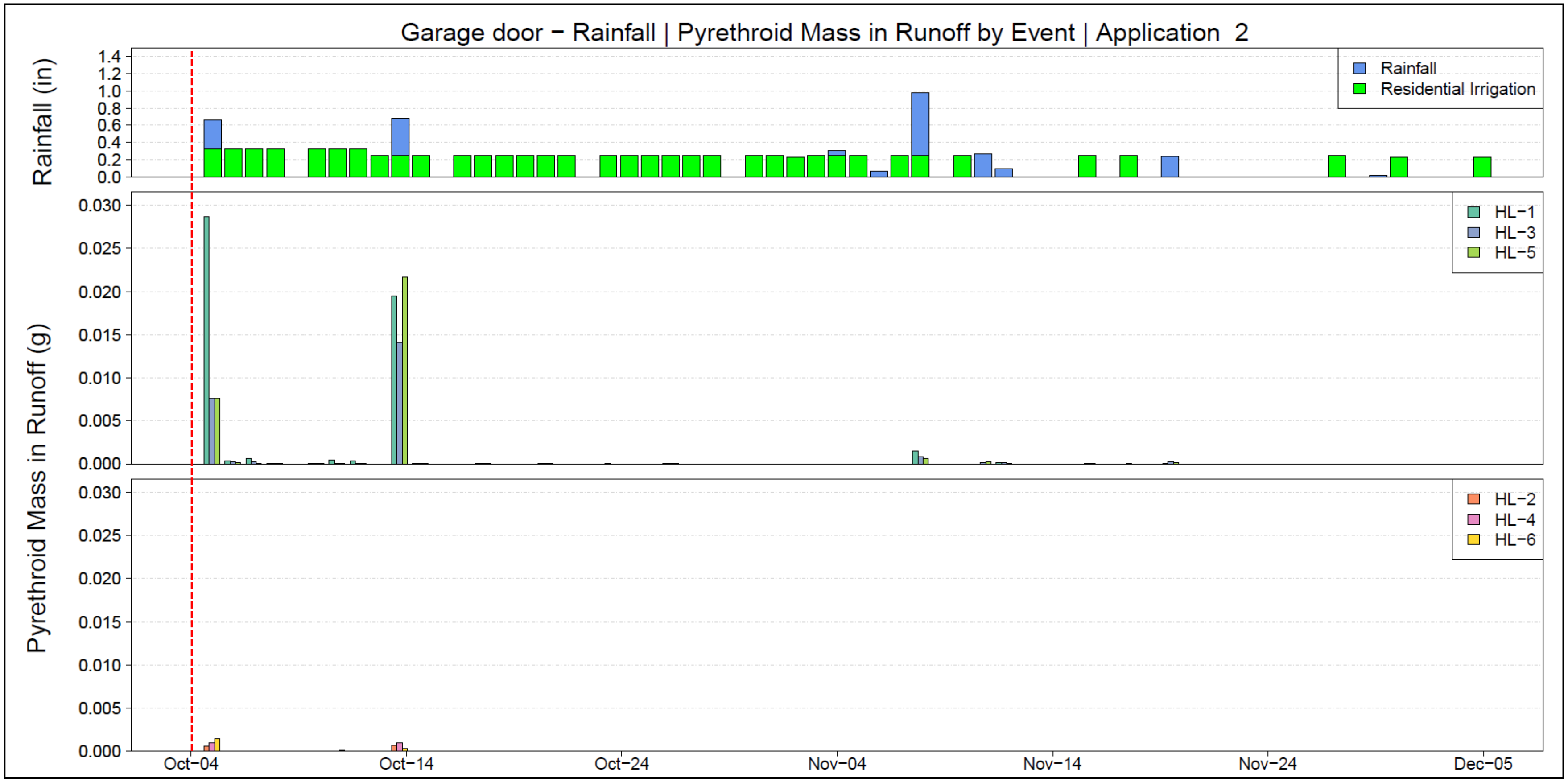

Figure S7-8. Runoff losses from the garage door and adjacent walls by event for each of the six house plots between the second and third sets of applications. The second application event occurred on October 4, 2011, as denoted by the vertical dashed line.

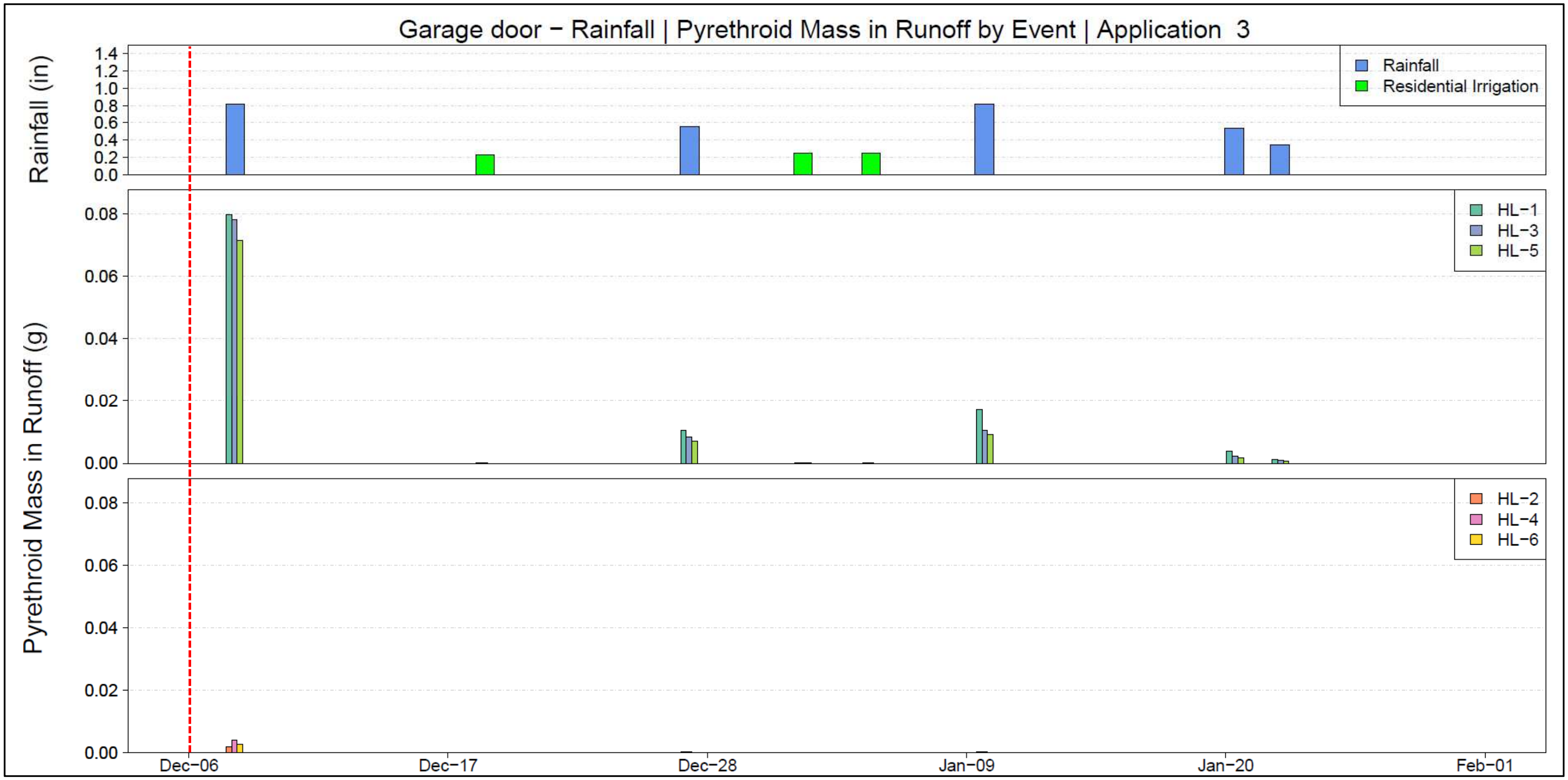

Figure S7-9. Runoff losses from the garage door and adjacent walls by event for each of the six house plots between the third and fourth sets of applications. The third application event occurred on December 6, 2011, as denoted by the vertical red dashed line.

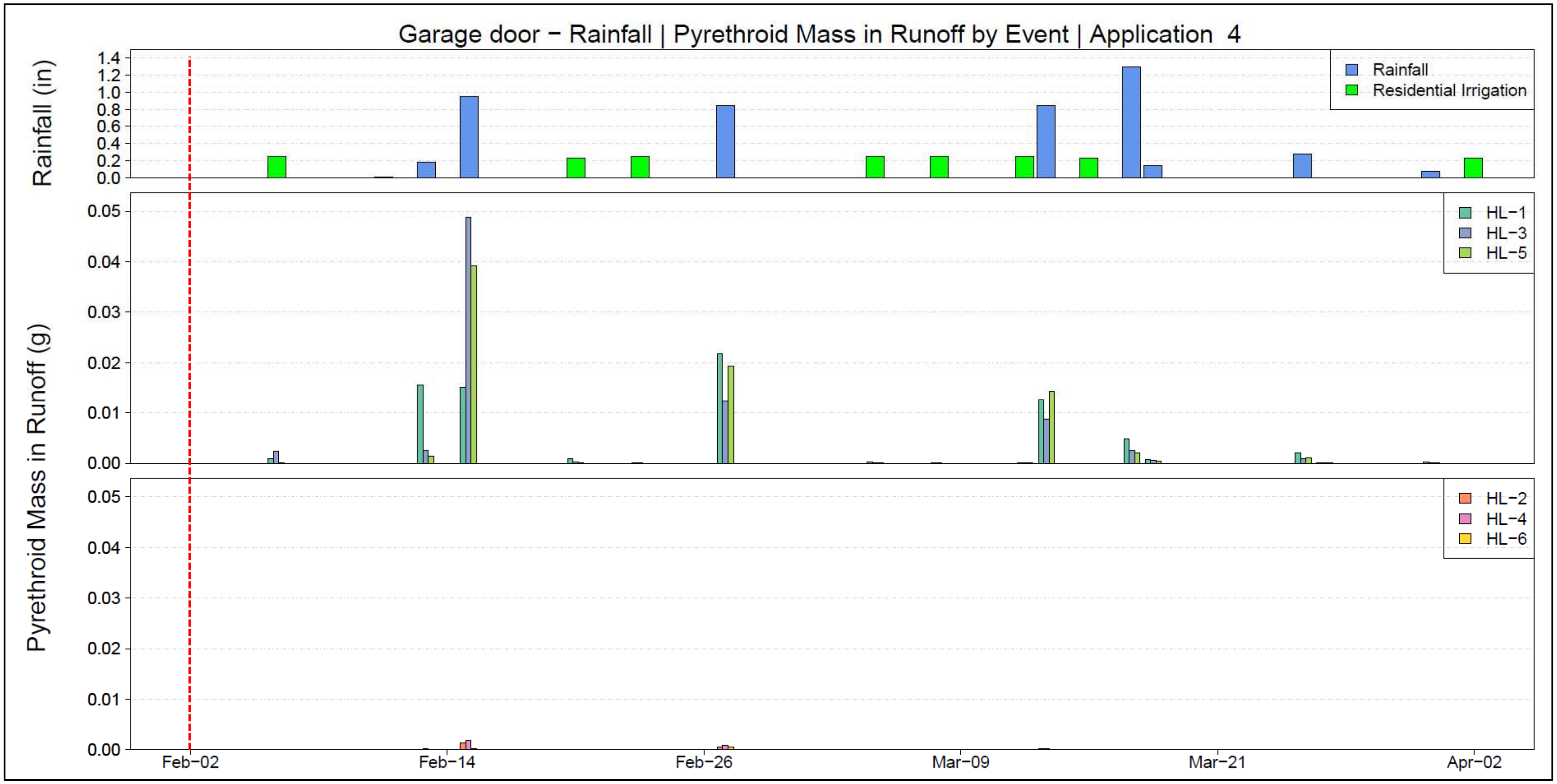

Figure S7-10. Runoff losses from the garage door and adjacent walls by event for each of the six house plots between the fourth and fifth sets of applications. The fourth application event occurred on February 2, 2012, as denoted by the vertical red dashed line.

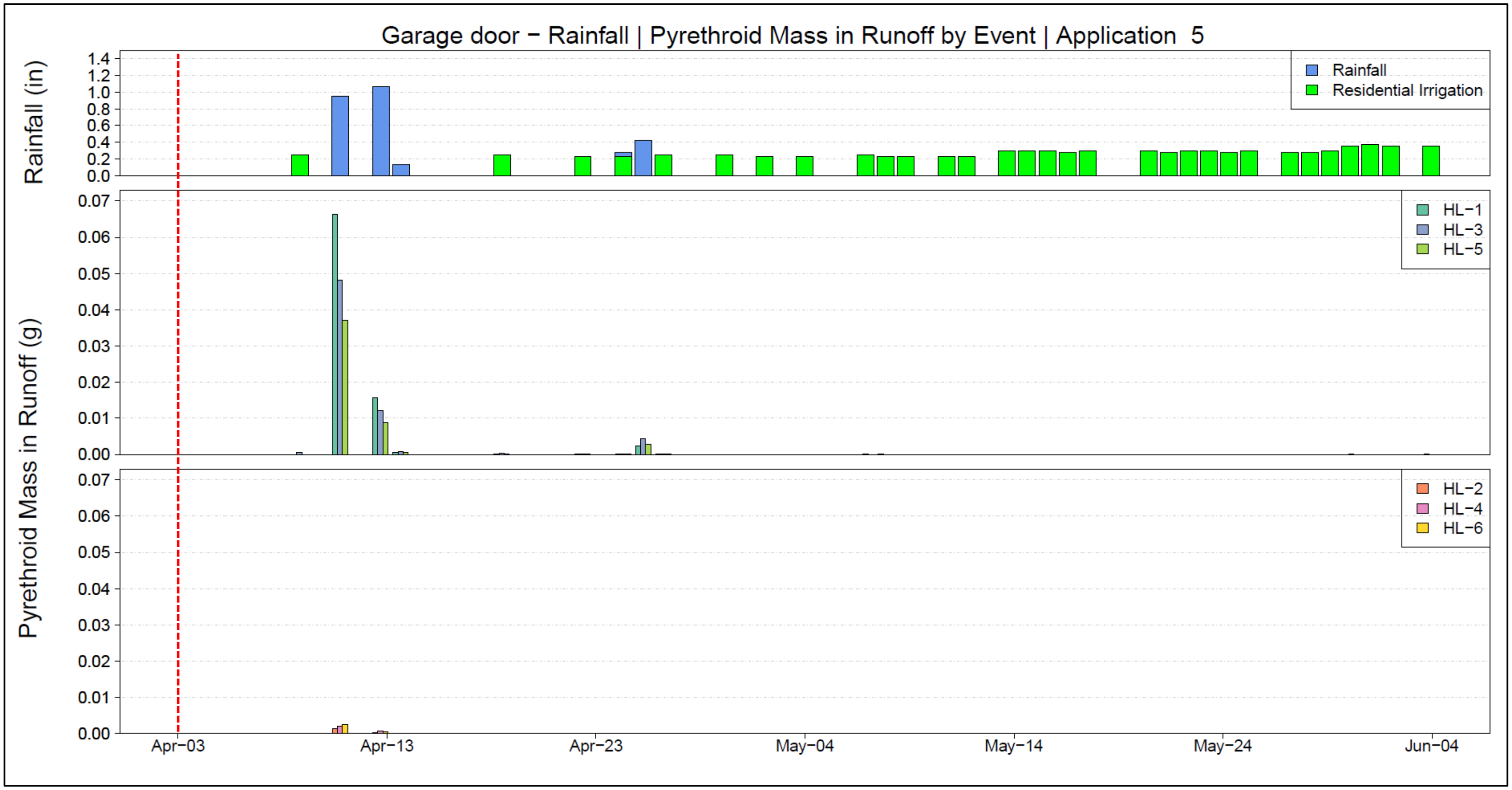

Figure S7-11. Runoff losses from the garage door and adjacent walls by event for each of the six house plots between the fifth and sixth sets of applications. The fifth application event occurred on April 3, 2012, as denoted by the vertical red dashed line.

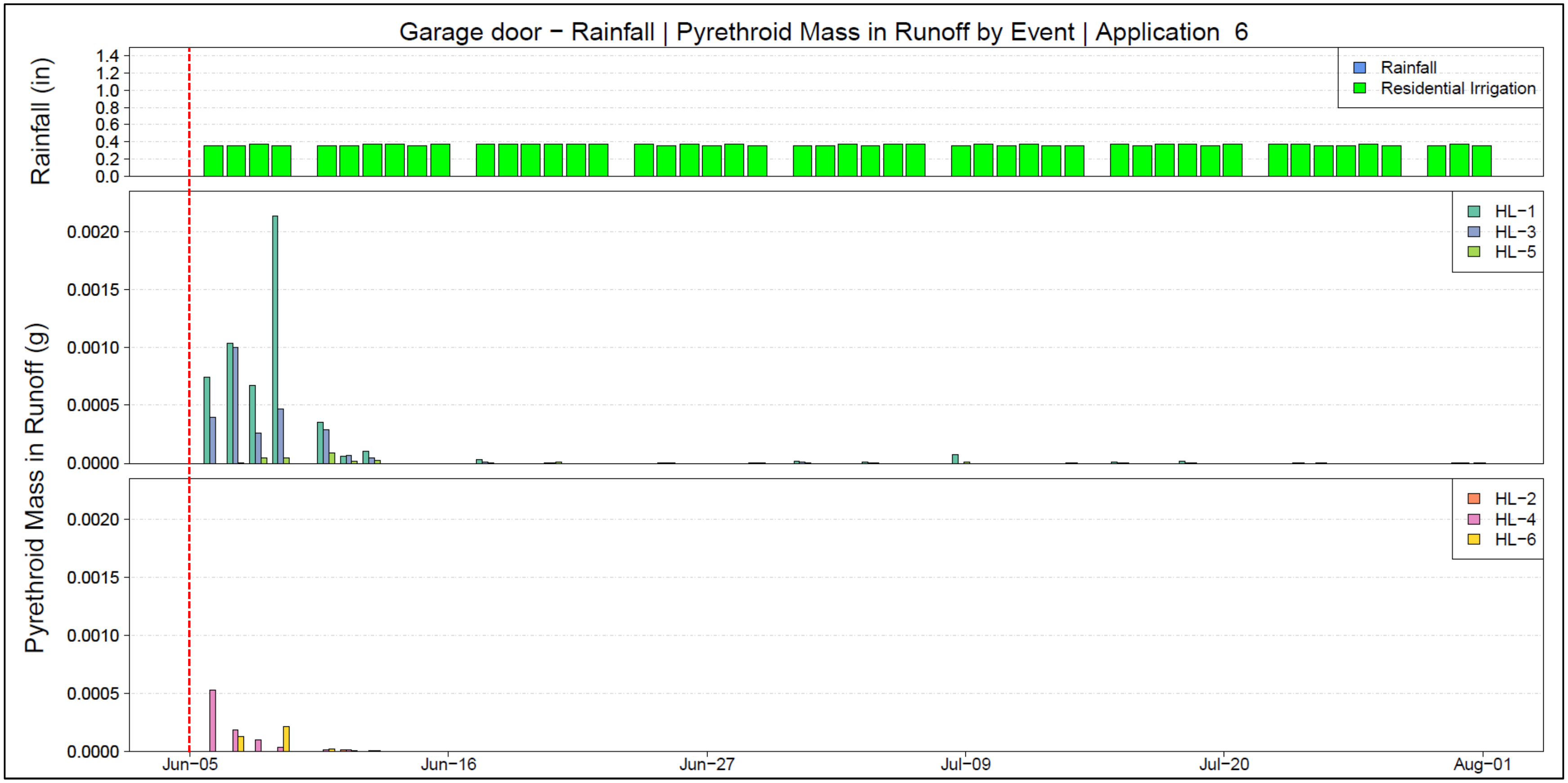

Figure S7-12. Runoff losses from the garage door and adjacent walls by event for each of the six house plots between the sixth application and the end of the study. The sixth application event occurred on June 5, 2012, as denoted by the vertical red dashed line.

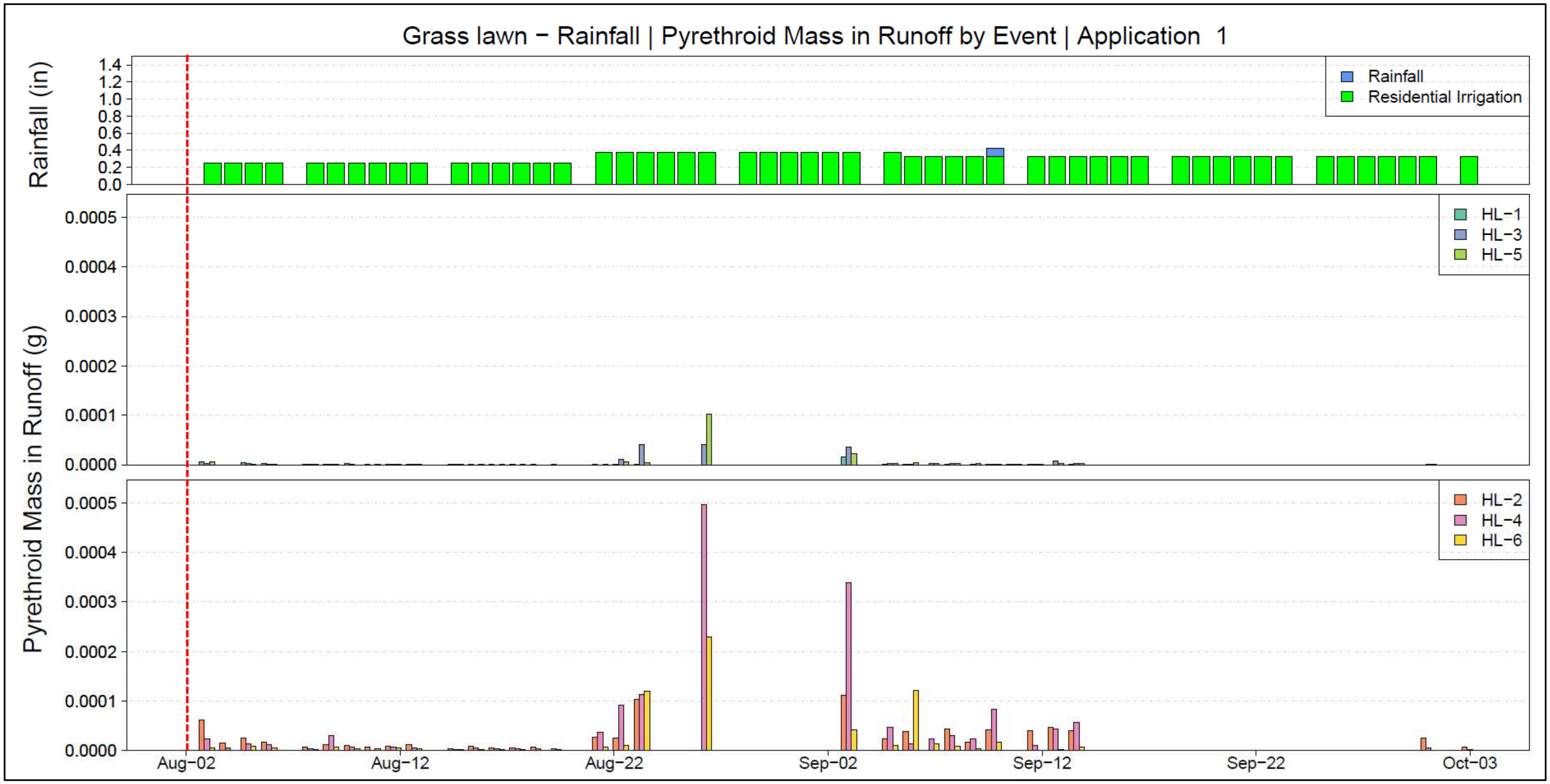

Figure S7-13. Runoff losses from the grass lawn by event for each of the six house plots between the first and second sets of applications. The first and only grass lawn application event occurred on August 2, 2011, as denoted by the vertical red dashed line.

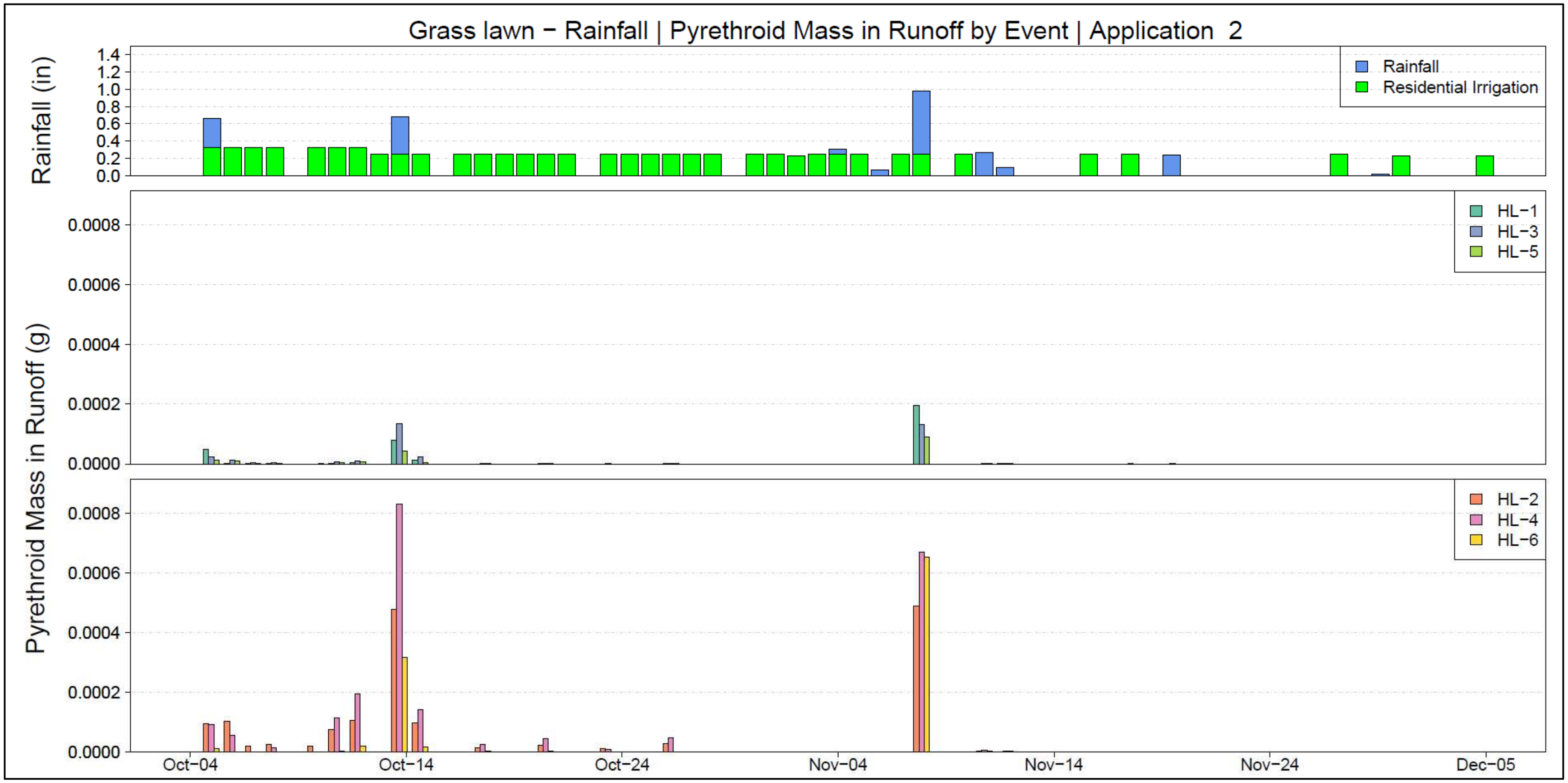

Figure S7-14. Runoff losses from the grass lawn by event for each of the six house plots between the second and third sets of applications (the lawn received an application only during the first set of applications).

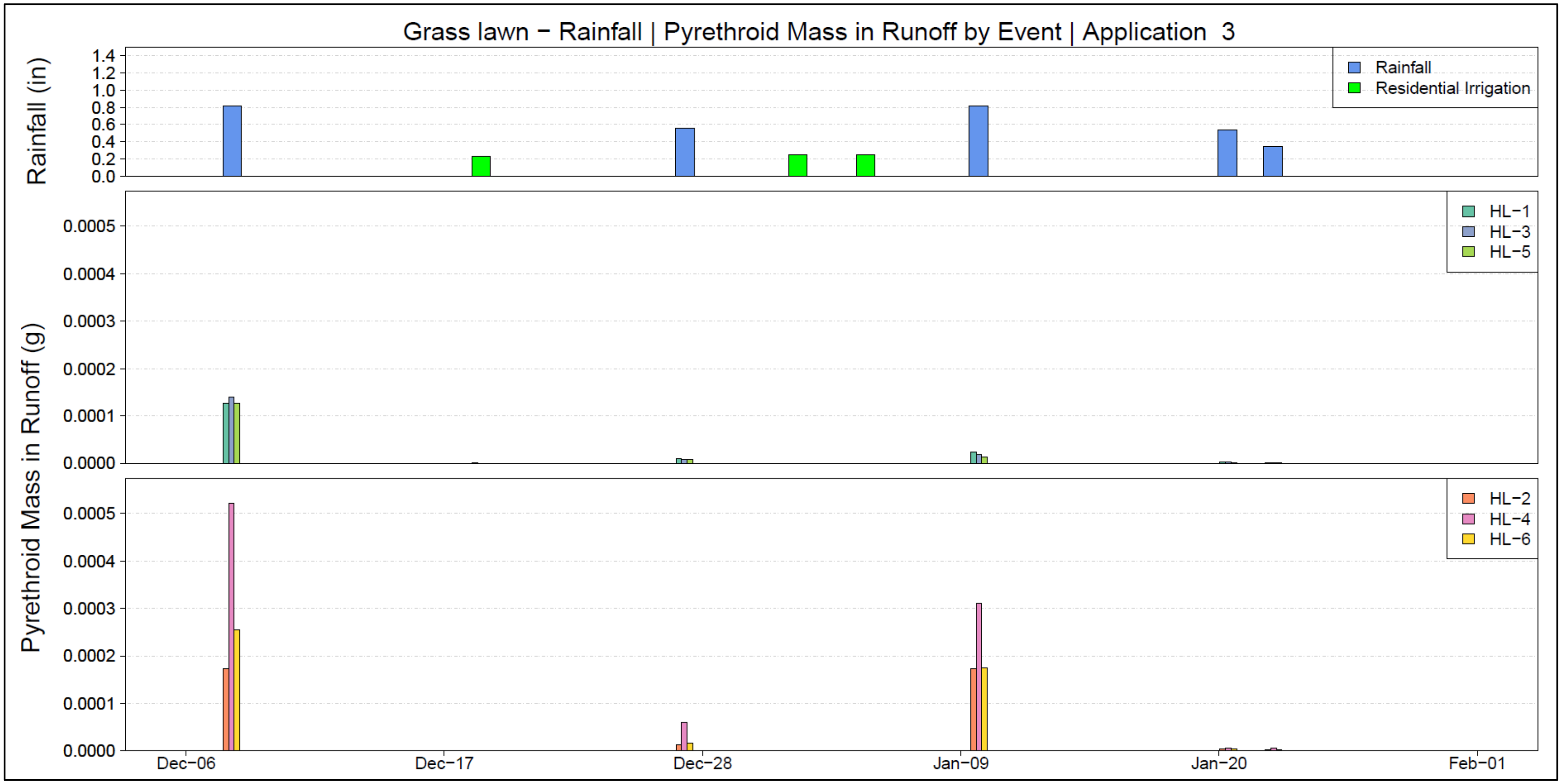

Figure S7-15. Runoff losses from the grass lawn by event for each of the six house plots between the third and fourth sets of applications (the lawn received an application only during the first set of applications).

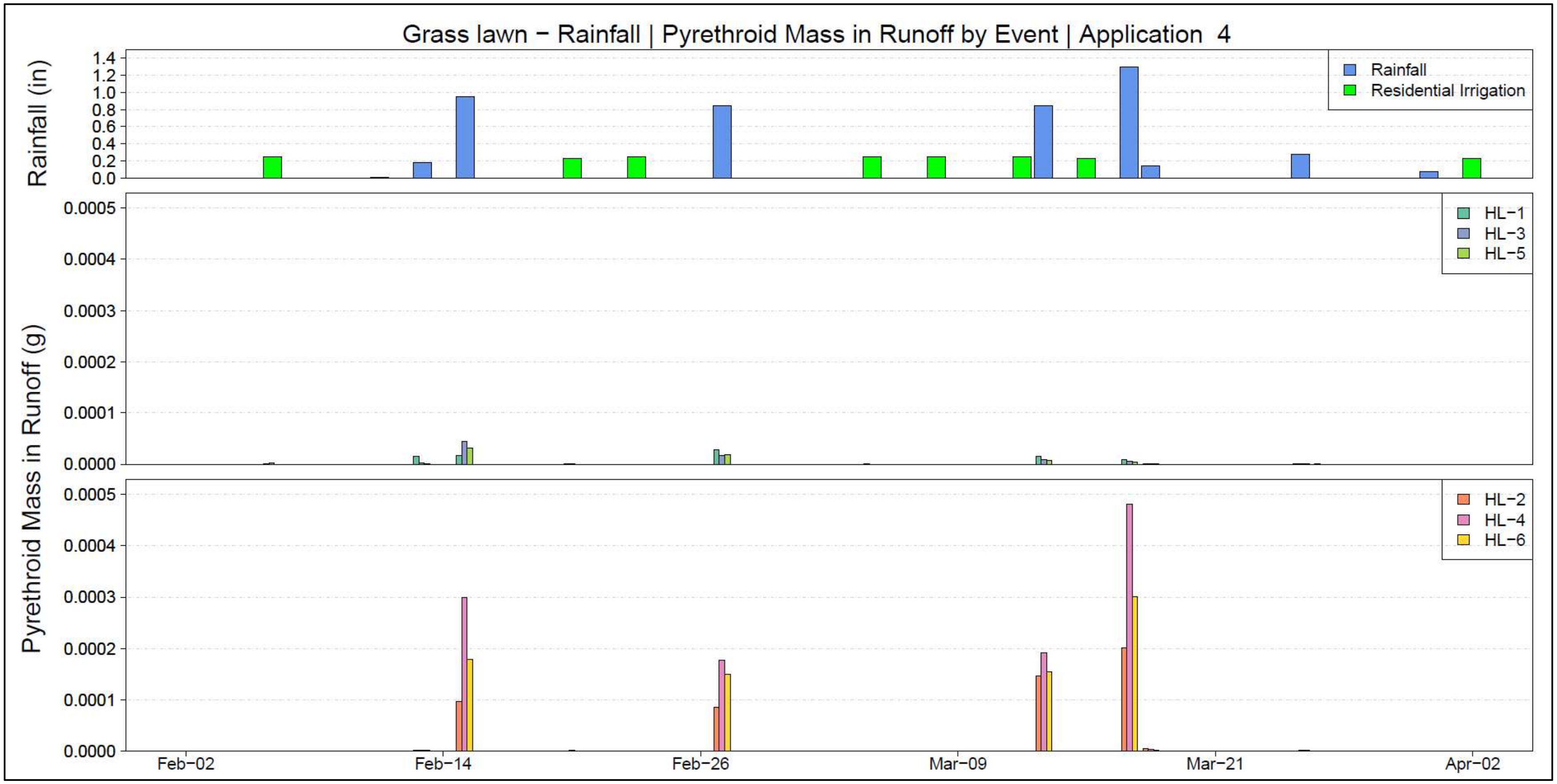

Figure S7-16. Runoff losses from the grass lawn by event for each of the six house plots between the fourth and fifth sets of applications (the lawn received an application only during the first set of applications).

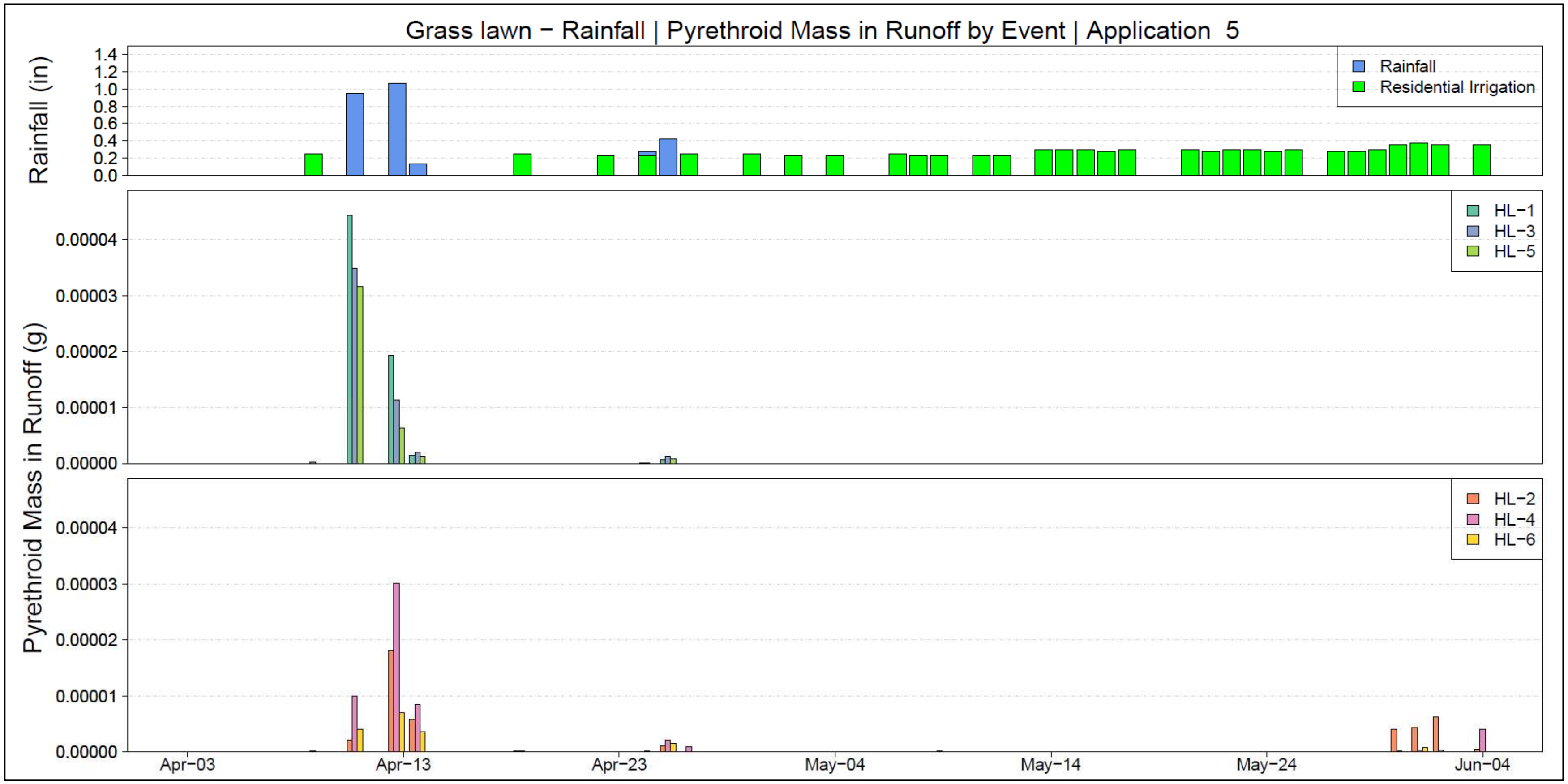

Figure S7-17. Runoff losses from the grass lawn by event for each of the six house plots between the fifth and sixth sets of applications (the lawn received an application only during the first set of applications).

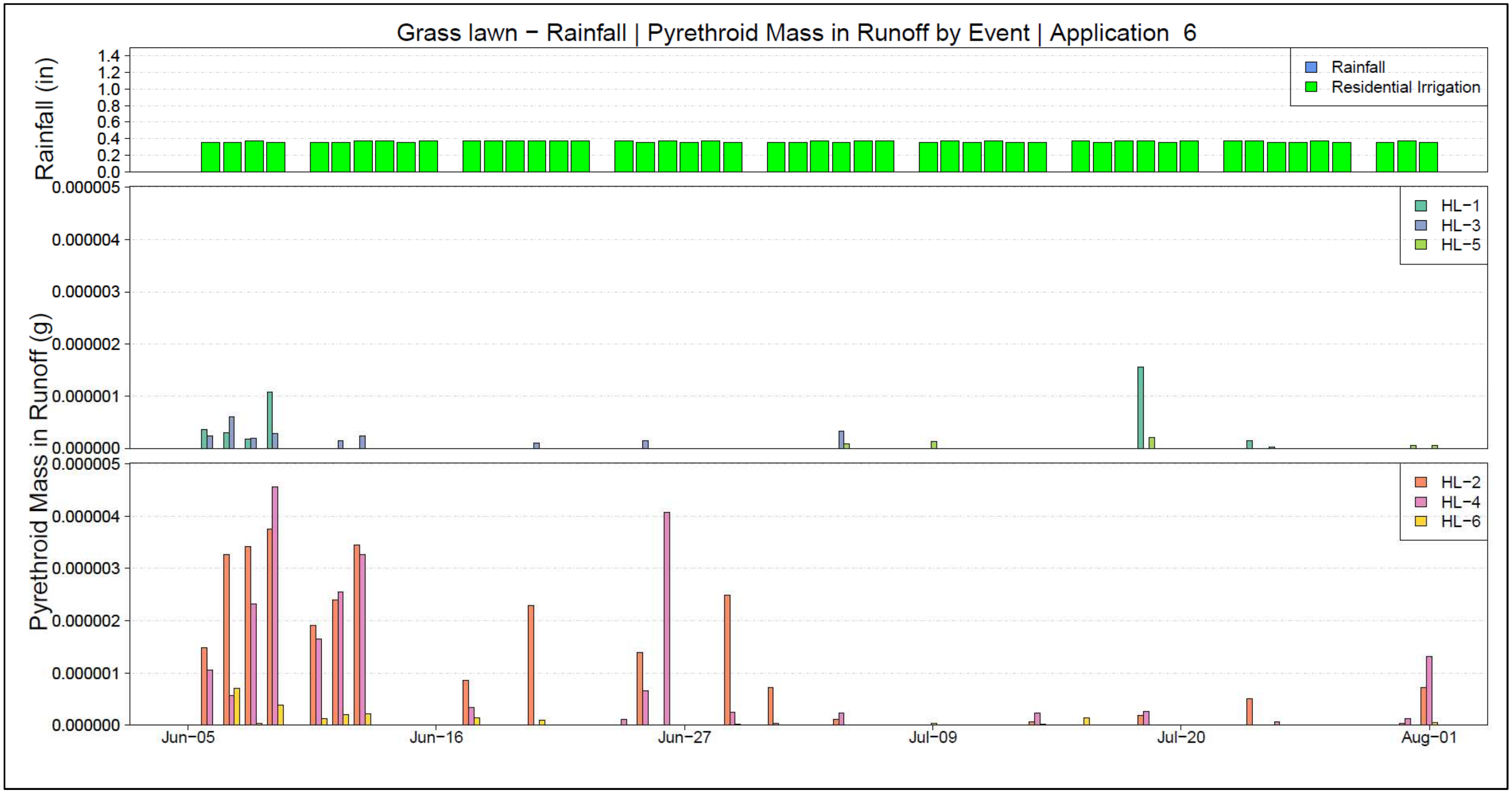

Figure S7-18. Runoff losses from the grass lawn by event for each of the six house plots between the sixth application and the end of the study (the lawn received an application only during the first set of applications).

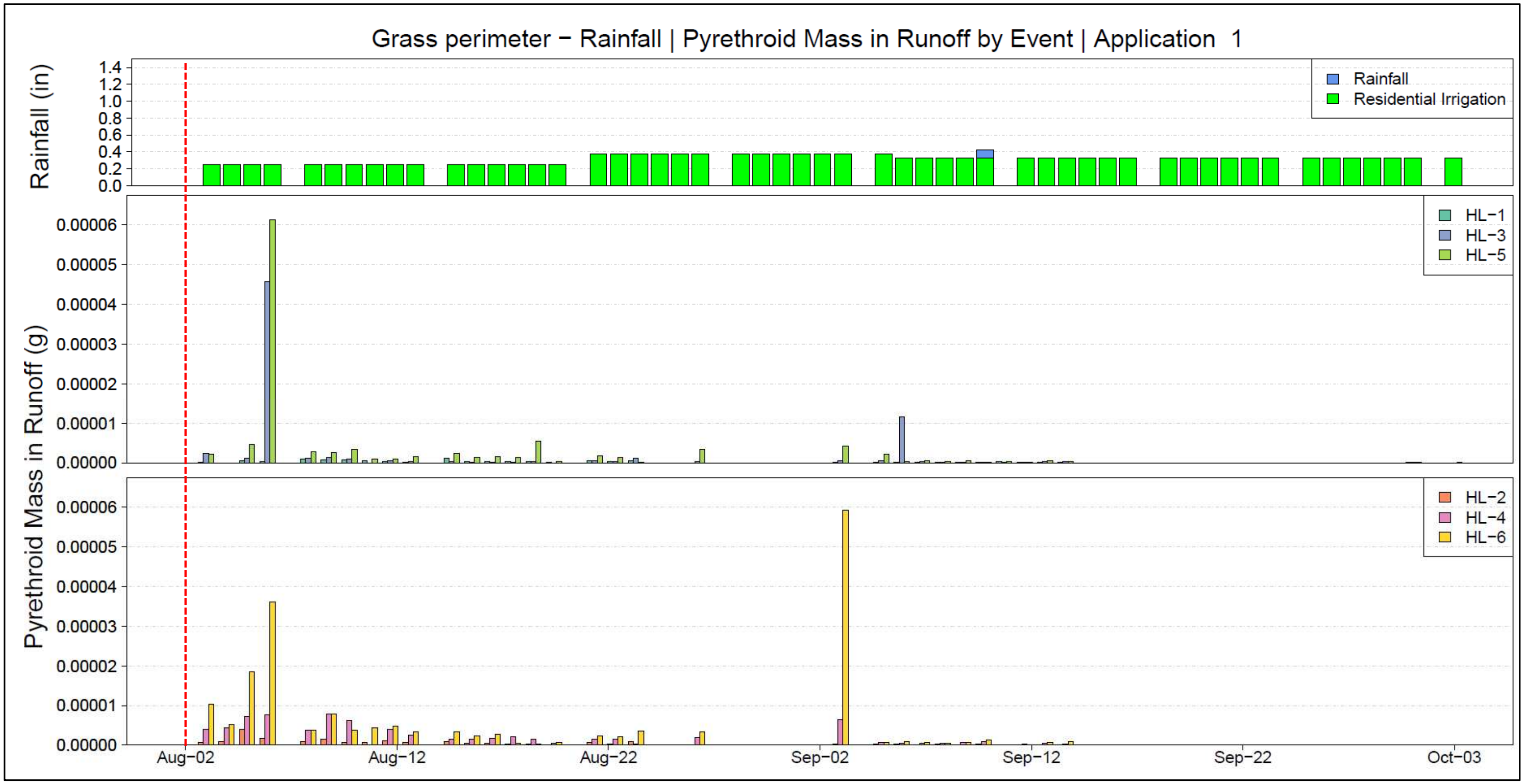

Figure S7-19. Runoff losses from the grass perimeter by event for each of the six house plots between the first and second sets of applications. The first application event occurred on August 2, 2011, as denoted by the vertical red dashed line.

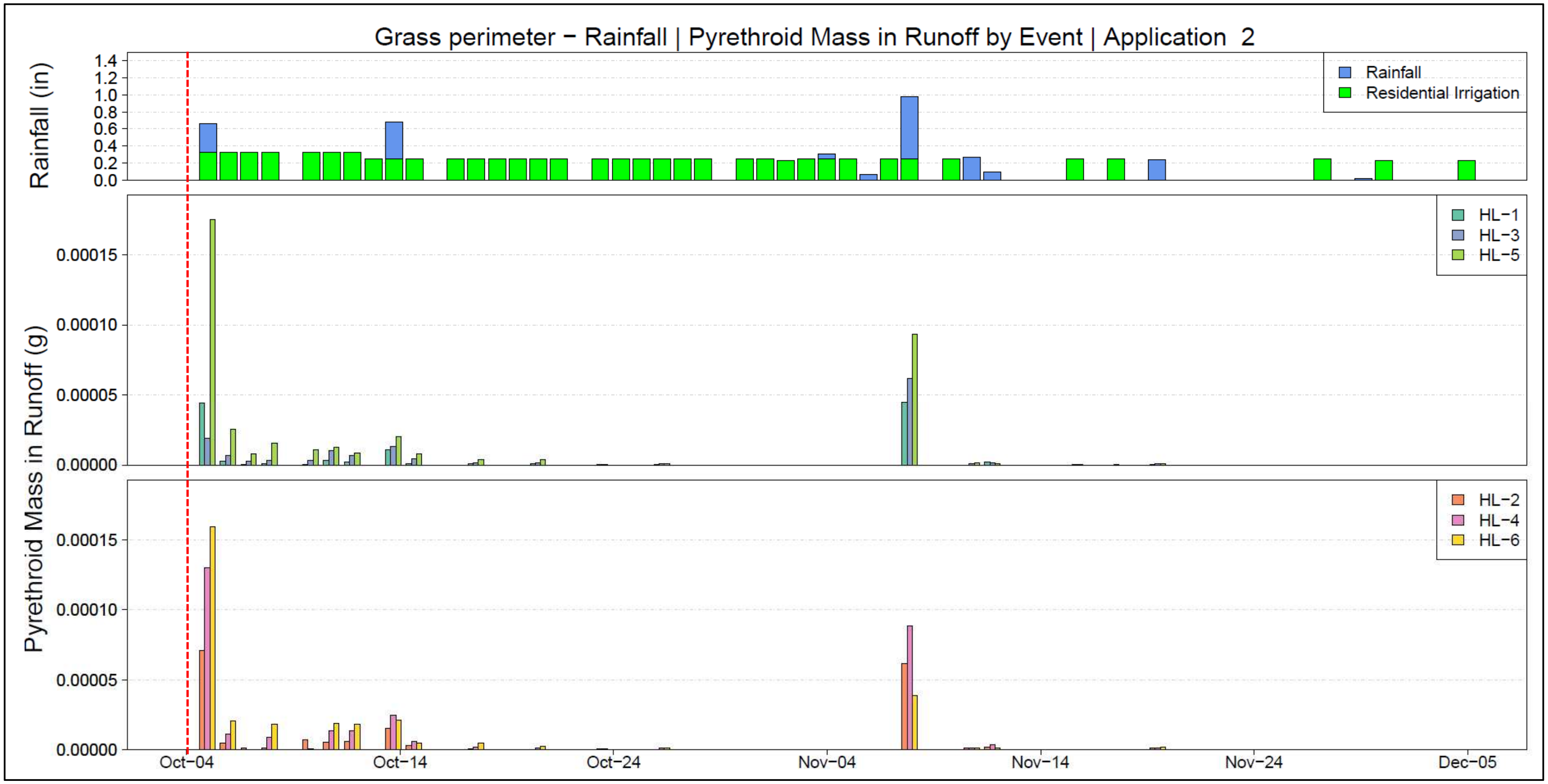

Figure S7-20. Runoff losses from the grass perimeter by event for each of the six house plots between the second and third sets of applications. The second application event occurred on October 4, 2011, as denoted by the vertical red dashed line.

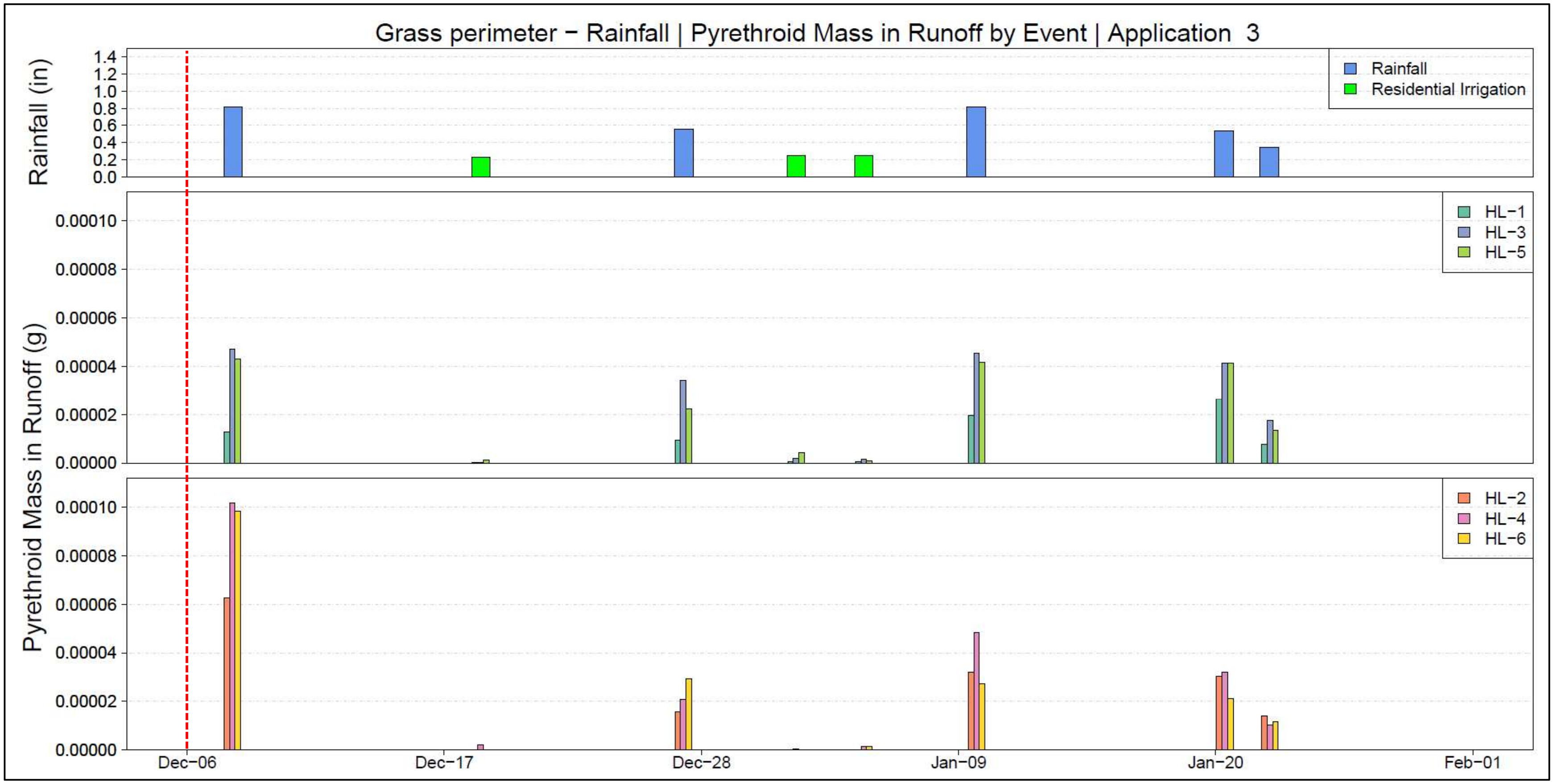

Figure S7-21. Runoff losses from the grass perimeter by event for each of the six house plots between the third and fourth sets of applications. The third application event occurred on December 6, 2011, as denoted by the vertical red dashed line.

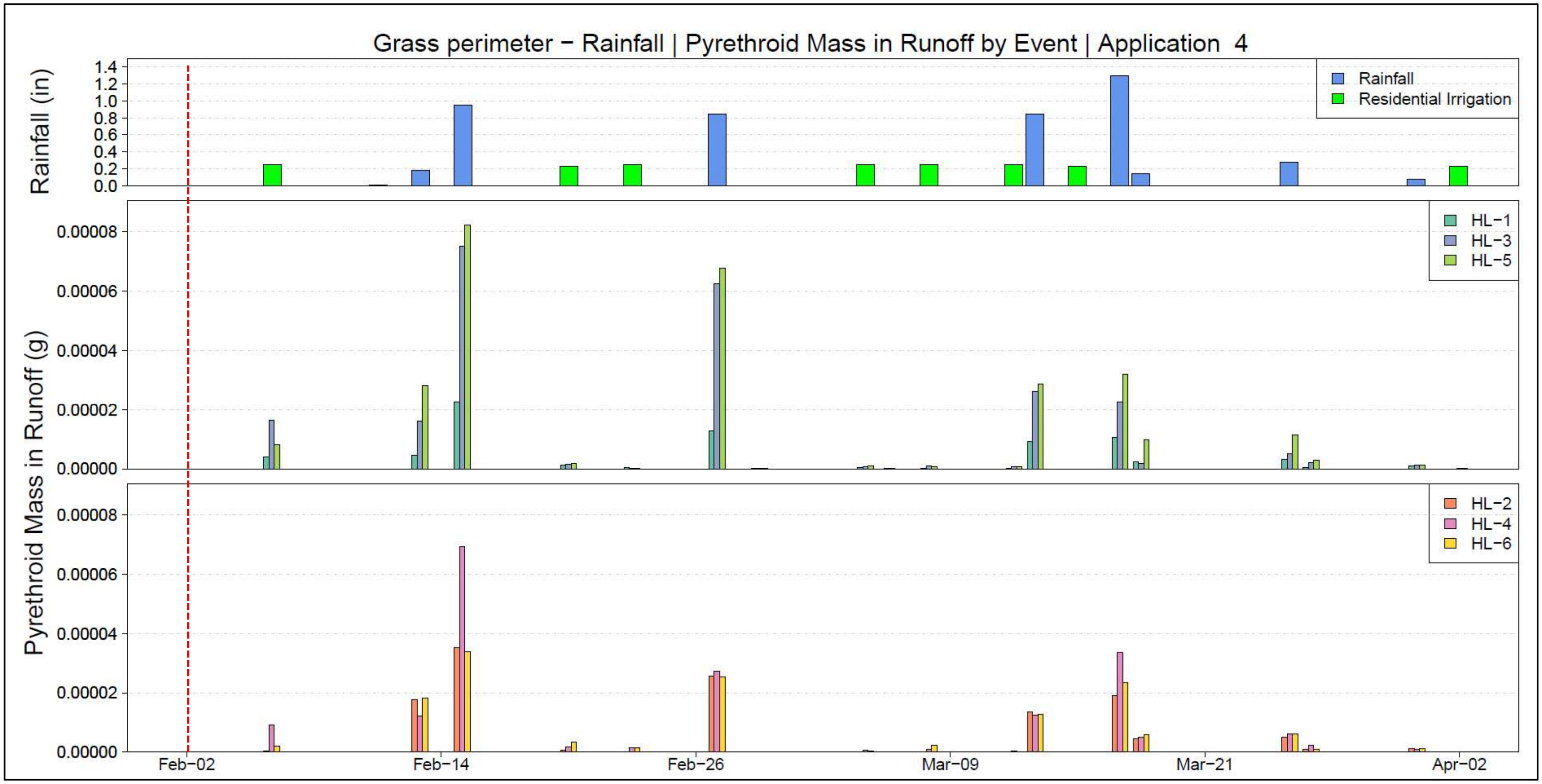

Figure S7-22. Runoff losses from the grass perimeter by event for each of the six house plots between the fourth and fifth sets of applications. The fourth application event occurred on February 2, 2012, as denoted by the vertical red dashed line.

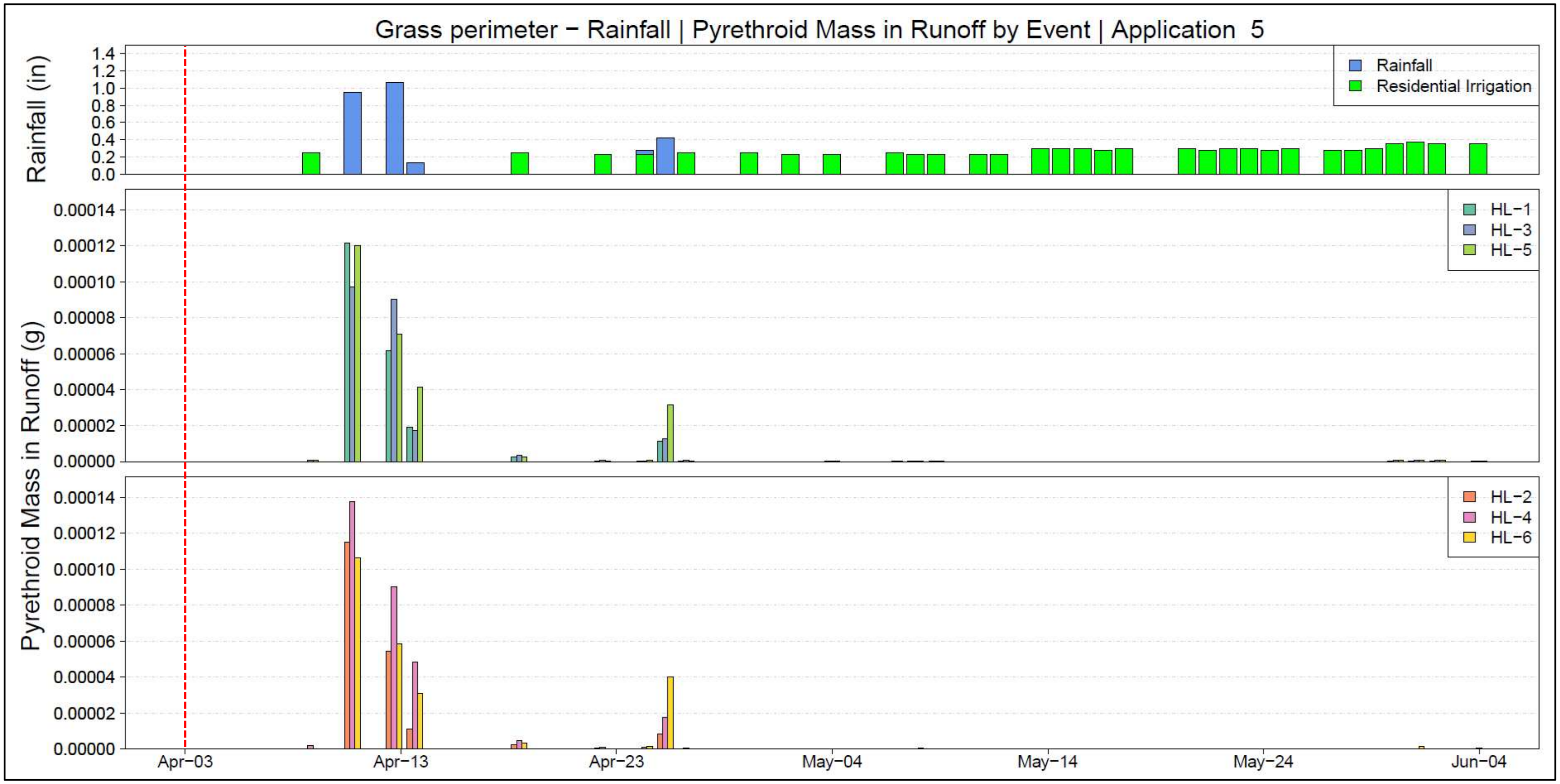

Figure S7-23. Runoff losses from the grass perimeter by event for each of the six house plots between the fifth and sixth sets of applications. The fifth application event occurred on April 3, 2012, as denoted by the vertical red dashed line.

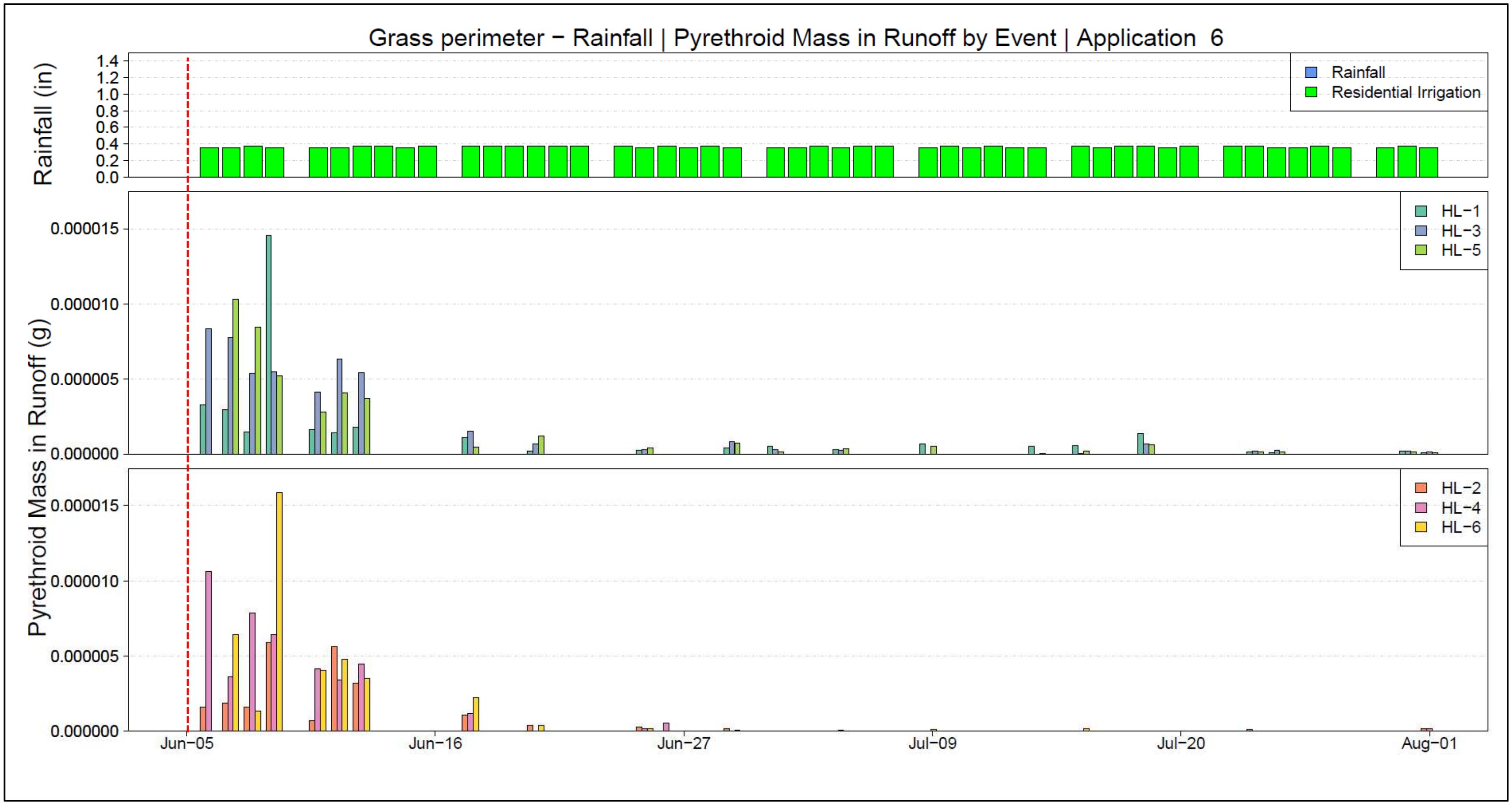

Figure S7-24. Runoff losses from the grass perimeter by event for each of the six house plots between the sixth application and the end of the study. The sixth application event occurred on June 5, 2012, as denoted by the vertical red dashed line.

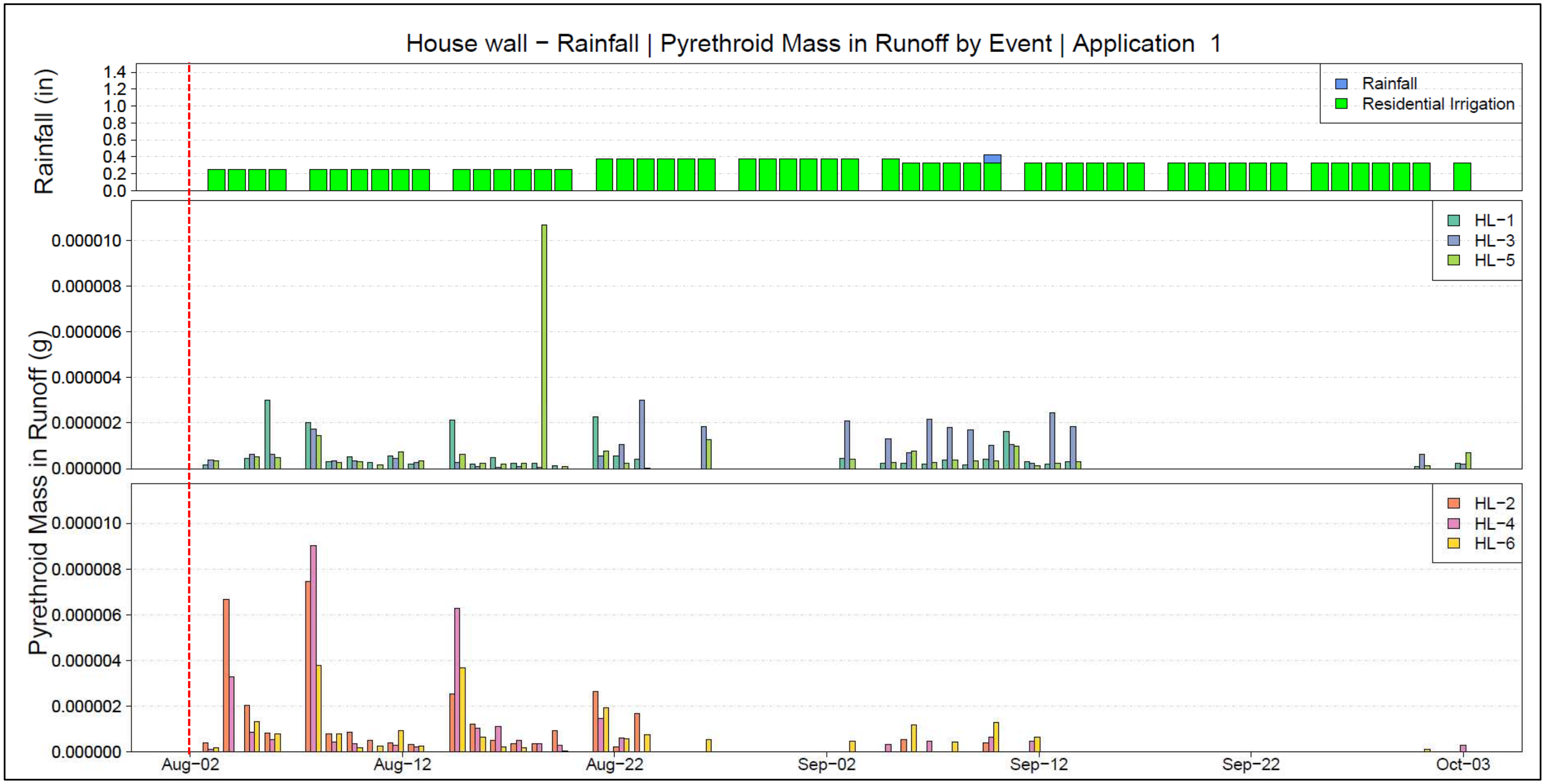

Figure S7-25. Runoff losses from the house wall by event for each of the six house plots between the first and second sets of applications. The first application event occurred on August 2, 2011, as denoted by the vertical red dashed line.

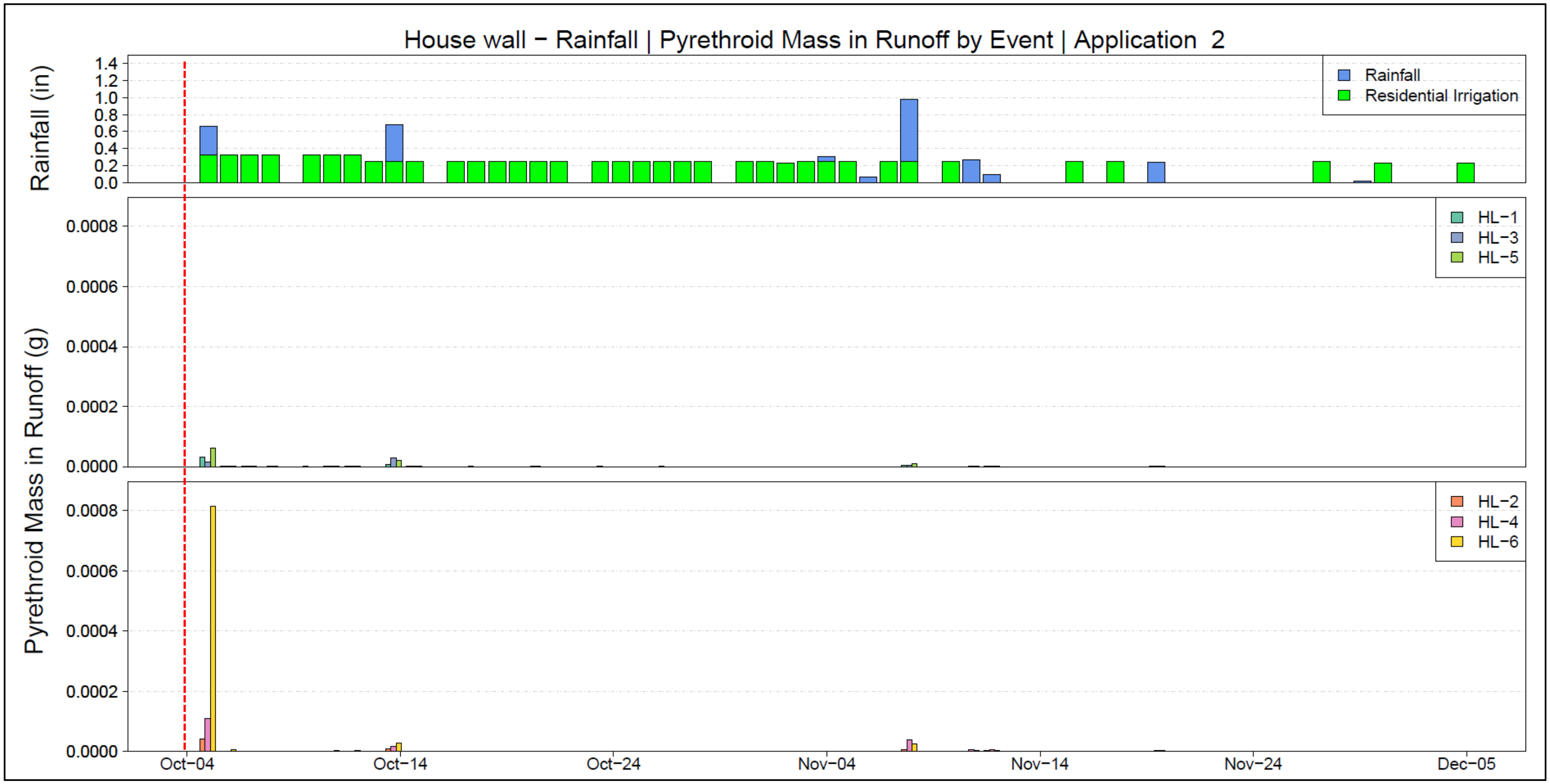

Figure S7-26. Runoff losses from the house wall by event for each of the six house plots between the second and third sets of applications. The second application event occurred on October 4, 2011, as denoted by the vertical red dashed line.

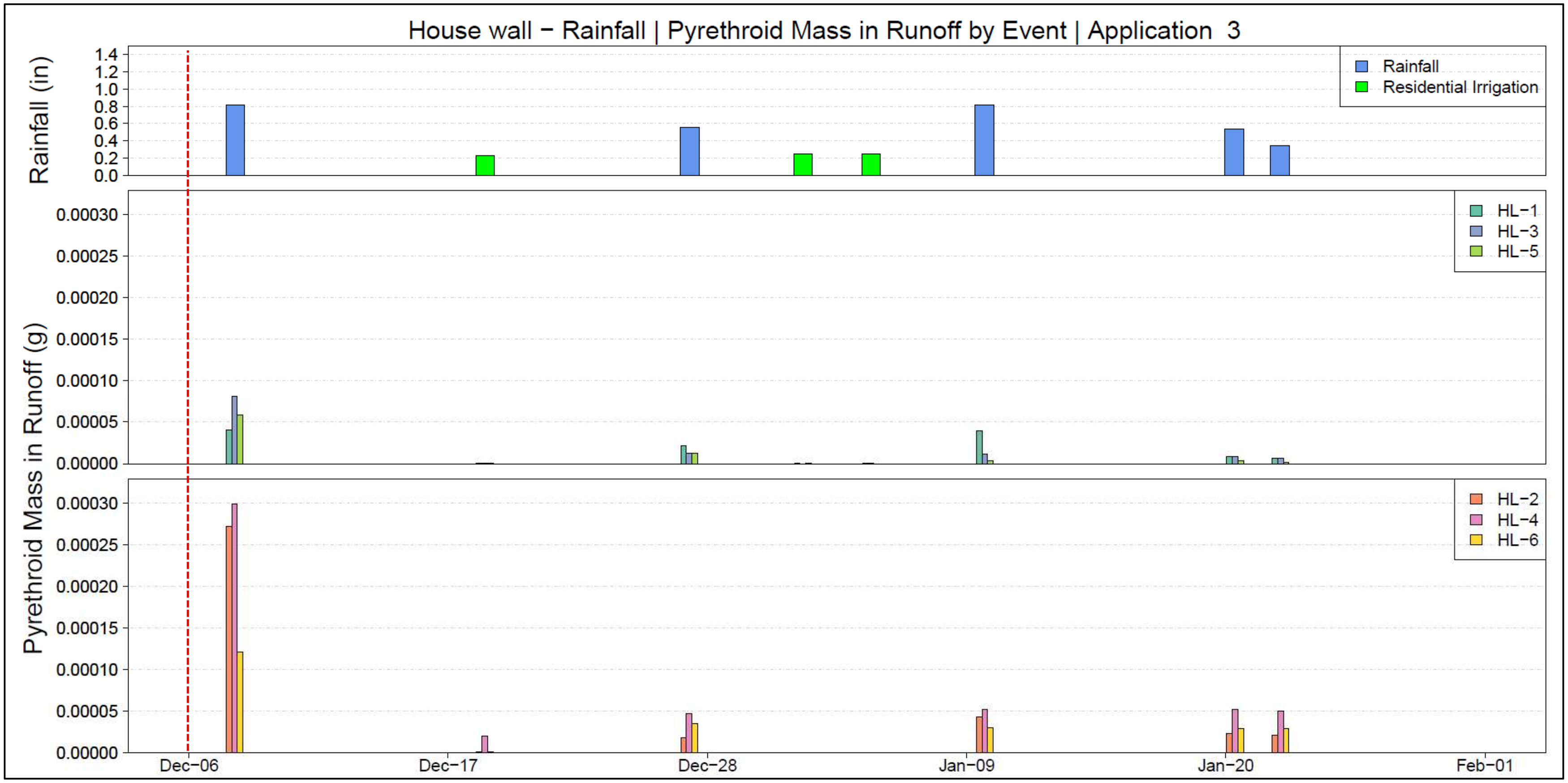

Figure S7-27. Runoff losses from the house wall by event for each of the six house plots between the third and fourth sets of applications. The third application event occurred on December 6, 2011, as denoted by the vertical red dashed line.

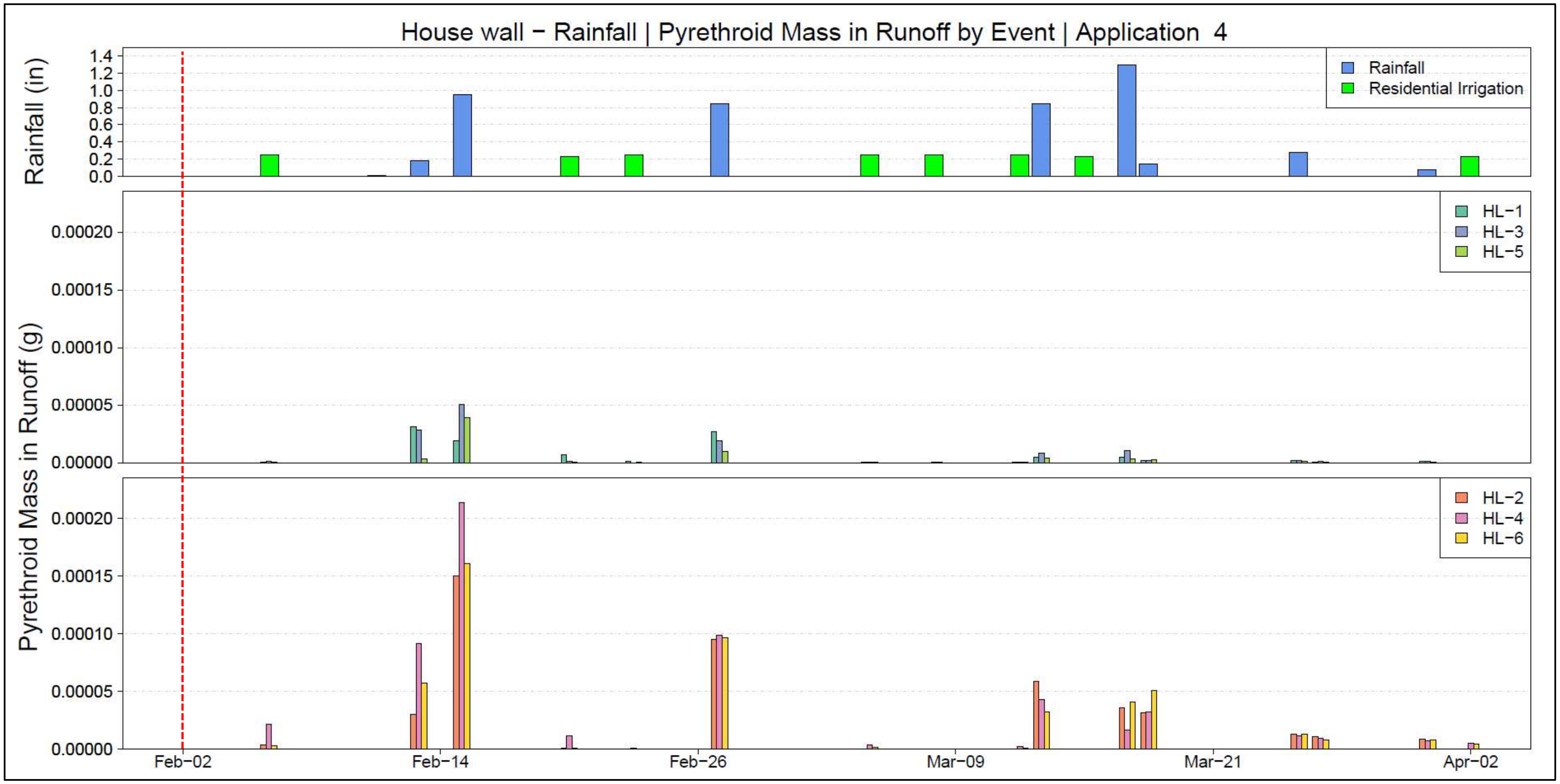

Figure S7-28. Runoff losses from the house wall by event for each of the six house plots between the fourth and fifth sets of applications. The fourth application event occurred on February 2, 2012, as denoted by the vertical red dashed line.

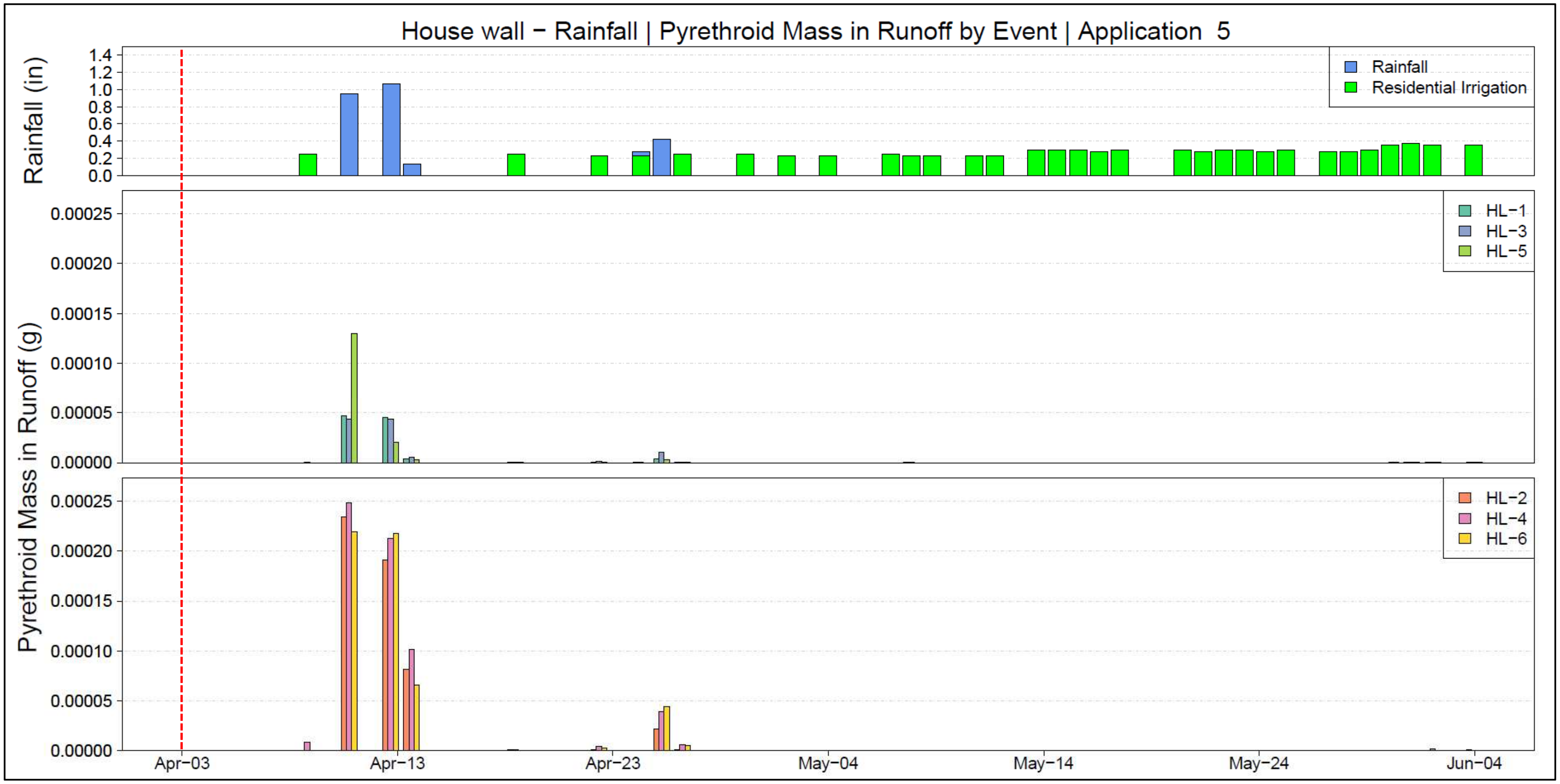

Figure S7-29. Runoff losses from the house wall by event for each of the six house plots between the fifth and sixth sets of applications. The fifth application event occurred on April 3, 2012, as denoted by the vertical red dashed line.

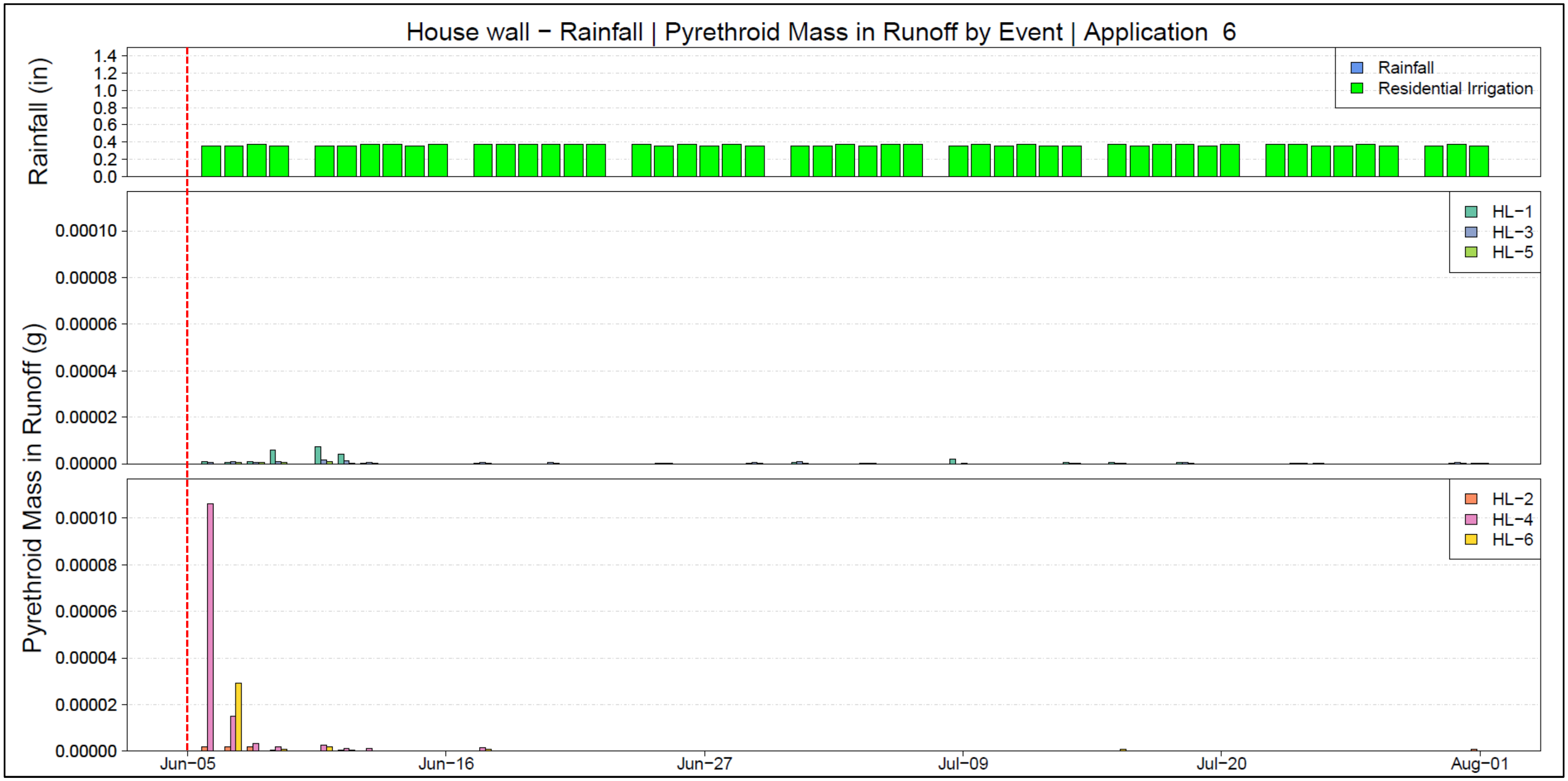

Figure S7-30. Runoff losses from the house wall by event for each of the six house plots between the sixth application and the end of the study. The sixth application event occurred on June 5, 2012, as denoted by the vertical red dashed line.
